# More Positive or More Negative? Metagenomic Analysis Reveals Roles of Virome in Human Disease-Related Gut Microbiome

**DOI:** 10.3389/fcimb.2022.846063

**Published:** 2022-04-12

**Authors:** Mo Li, Chunhui Wang, Qian Guo, Congmin Xu, Zhongjie Xie, Jie Tan, Shufang Wu, Peihong Wang, Jinyuan Guo, Zhencheng Fang, Shiwei Zhu, Liping Duan, Xiaoqing Jiang, Huaiqiu Zhu

**Affiliations:** ^1^Peking University-Tsinghua University-National Institute of Biological Sciences (PTN) Joint Ph.D. Program, School of Life Sciences, Peking University, Beijing, China; ^2^Department of Biomedical Engineering, College of Future Technology, Peking University, Beijing, China; ^3^Center for Quantitative Biology, Peking University, Beijing, China; ^4^Department of Gastroenterology, Peking University Third Hospital, Beijing, China; ^5^Institute of Medical Technology, Peking University Health Science Center, Beijing, China

**Keywords:** virus, gut microbiome, health and disease, microbial community, viral database

## Abstract

Viruses are increasingly viewed as vital components of the human gut microbiota, while their roles in health and diseases remain incompletely understood. Here, we first sequenced and analyzed the 37 metagenomic and 18 host metabolomic samples related to irritable bowel syndrome (IBS) and found that some shifted viruses between IBS and controls covaried with shifted bacteria and metabolites. Especially, phages that infect beneficial lactic acid bacteria depleted in IBS covaried with their hosts. We also retrieved public whole-genome metagenomic datasets of another four diseases (type 2 diabetes, Crohn’s disease, colorectal cancer, and liver cirrhosis), totaling 438 samples including IBS, and performed uniform analysis of the gut viruses in diseases. By constructing disease-specific co-occurrence networks, we found viruses actively interacting with bacteria, negatively correlated with possible dysbiosis-related and inflammation-mediating bacteria, increasing the connectivity between bacteria modules, and contributing to the robustness of the networks. Functional enrichment analysis showed that phages interact with bacteria through predation or expressing genes involved in the transporter and secretion system, metabolic enzymes, *etc*. We further built a viral database to facilitate systematic functional classification and explored the functions of viral genes on interacting with bacteria. Our analyses provided a systematic view of the gut virome in the disease-related microbial community and suggested possible positive roles of viruses concerning gut health.

## Introduction

The gut viruses have received increasing attention due to our recent comprehension that the human gut microbiota is a dense and taxonomically diverse consortium of microorganisms while containing all four superkingdoms, *Bacteria*, *Archaea*, *Eukarya*, and *Viruses*. Phages, known as viruses of bacteria, have been found to play notable roles in the predation of bacteria and horizontal gene transfer (Shkoporov and Hill, 2019). Moreover, evidence from the experimental study shows that phages demonstrate cascading effects on microbiota species and can modulate metabolites, further affecting mammalian hosts (Hsu et al., 2019). As for eukaryotic viruses, the direct interaction with bacteria may facilitate viral infection when viruses can infect humans (Berger and Mainou, 2018). Notably, the eukaryotic viruses also demonstrated complex interactions with bacteria, and much of the mechanisms are still unknown (Almand et al., 2017; Berger and Mainou, 2018).

For the human gut, regardless of the extensively studied relationships between diseases and bacteria, less attention has been paid to viruses. Being the total collection of viruses within the gut microbiota, the gut virome is suggested to infect human cells as described above, as well as other microbes such as bacteria. Studies have shown that double-stranded DNA phage, the *Caudovirales* order, is the major human gut virus. Single-stranded phage, the *Microviridae* order, is also abundant in some individuals (Manrique et al., 2016; Shkoporov et al., 2019). Besides, substantial amounts of gut phages exist in the bacteria genome in the form of prophages (Silveira and Rohwer, 2016; Sutton and Hill, 2019). However, the majority of the viruses in gut microbiota are uncharacterized yet, and their roles in shaping the gut microbial community and affecting human health remain poorly understood (Shkoporov et al., 2019). Although many studies have reported the shifted gut virome in acute and chronic diseases, such as severe acute malnutrition (Reyes et al., 2015), irritable bowel syndrome (IBS) (Coughlan et al., 2021; Mihindukulasuriya et al., 2021), Crohn’s disease (CD) (Pérez-Brocal et al., 2013; Norman et al., 2015; Clooney et al., 2019), colorectal cancer (CRC) (Gao et al., 2021), ulcerative colitis, and type 2 diabetes (T2D) (Norman et al., 2015; Ma et al., 2018; Clooney et al., 2019), these alterations of viral elements in individual cases are still insufficient to understand the specific roles of viruses in diseases systematically. Besides, studies have shown that altered co-abundance relationships between bacteria and topological distortion of the network structure occurred in the disease-related gut microbiome (Baldassano and Bassett, 2016; Chen et al., 2020). However, these studies overlooked viruses that might push the network changes. At present, metagenomic sequencing is one of the methods to study virome (Ma et al., 2018; Garmaeva et al., 2019), which takes prophages that exist in bacteria genomes into consideration. Utilizing metagenomes also makes the quantification of viruses and bacteria on the same scale and thus convenient to construct interaction networks. To sum up, metagenomic studies that systematically characterize viruses in diseases and their relationships with bacteria are still lacking.

Here, we conducted an exploratory analysis of virome from gut metagenomic sequencing datasets of five diseases [IBS, T2D, CD, CRC, and liver cirrhosis (LC)] to get more profound insights into the roles that viruses play in the gut microbial community of health and diseases. We started from the analyses of IBS datasets from the recruited subjects, including 22 cases and 15 healthy controls (9 of each group have the host serum metabolomics data). We collected 401 disease-control gut metagenomic public data of the other four diseases (Qin et al., 2012; Qin et al., 2014; Lewis et al., 2015; Yu et al., 2017) (438 metagenomic data in total) to analyze gut viruses in multiple diseases. With a well-designed metagenomic sequence analysis and viral gene identification pipeline, we found there were shifts in viral composition, which showed consistency with the shifts in bacteria and metabolome between IBS patients and healthy controls. Significantly, the shifted viruses included phages that infect several lactic acid bacteria depleted in the IBS group. By further constructing and analyzing the disease-specific networks, we found that viruses actively interacted with bacteria in both diseased and healthy guts. Moreover, viruses showed a significant trend of more negatively correlating with dysbiosis-related bacteria and inflammation-related bacteria such as *Proteobacteria* and *Bacteroidetes* in multiple diseases, indicating possible inhibitory effects against these bacteria. Besides, we found a list of key viruses that were of high centrality and contributed most to the whole communication of microbes in the pan-network and shortened path length among major short-chain fatty acid-producing bacteria. Lastly, we characterized the functions of viral genes by manual categorization of family annotations and built a database named VirGenFunD (gut Viral Genes and Functional classification Database) for the detected viral sequences (available at http://cqb.pku.edu.cn/ZhuLab/VirGenFunD/, or https://yjiang724.github.io/VirGenFunD/). The functional annotations of VirGenFunD thus doubled the number of the known function categories. These results presented a landscape of viruses in the disease-related gut microbial network and provided insights to a better understanding of the human gut microbiome and potential treatments of diseases.

## Materials and Methods

### Metagenomic Sample Description

This study includes five metagenomic datasets, including IBS datasets sequenced from recruited subjects and four other datasets from the public database. The dataset of 22 IBS patients and 15 healthy controls have been described in our previous paper (Xu et al., 2020). Briefly, these subjects were recruited at the Outpatient Department of Gastroenterology of Peking University Third Hospital. The studies involving human participants were reviewed and approved by the Ethics Committee of Peking University People’s Hospital (No.2017PHB105-01). The participants provided their written informed consent to participate in this study. IBS patients should meet the standard of the Rome III criteria. Exclusion criteria included organic gastrointestinal or systemic diseases, use of antibiotics or antidepressants within a month, and use of probiotics, laxatives, or antidiarrheal drugs for more than 3 days during the previous 2 weeks. Among these individuals, nine cases and nine controls have corresponding metabolomic data (non-targeted metabolomics profiling on serums). Details of the sample collection, DNA sequencing, and metabolomic assay are documented in **Supplementary Methods** in **Supplementary Materials**.

Another four metagenomic published datasets were also obtained from studies related to the following diseases: T2D (Qin et al., 2012), CD (Lewis et al., 2015), CRC, and LC (Qin et al., 2014; Yu et al., 2017). The selection of these four diseases was mainly in consideration of metabolic or bowel dysfunctional diseases and data availability when we performed the research. Sample information is included in **Table S4**. Except for one CD dataset which is an American cohort, all are Chinese cohorts.

### Data Processing

We began with the raw reads and processed them in a uniform pipeline. Reads were first quality controlled by prinseq-lite, with arguments -ns_max_p 10 and -min_qual_mean 25. Then, human sequences were removed by mapping reads to human reference genome GRCh38 with bowtie2 using the argument -very-fast (Langmead and Salzberg, 2012). The remaining reads were considered as clean reads. To assure the quality of these samples, only samples of clean reads fastq files larger than 2 Gb and contig N50 lengths longer than 1 kb were included, which resulted in 27 cases versus 31 controls in the T2D dataset, 148 cases versus 18 controls in the CD dataset, 52 cases versus 51 controls in the CRC dataset, and 40 cases versus 34 controls in the LC dataset. The 37 sequenced samples in the IBS dataset all met the sample quality standard.

### Viral Gene Identification and Bacteria Taxonomic Annotation

**Figure 1:**
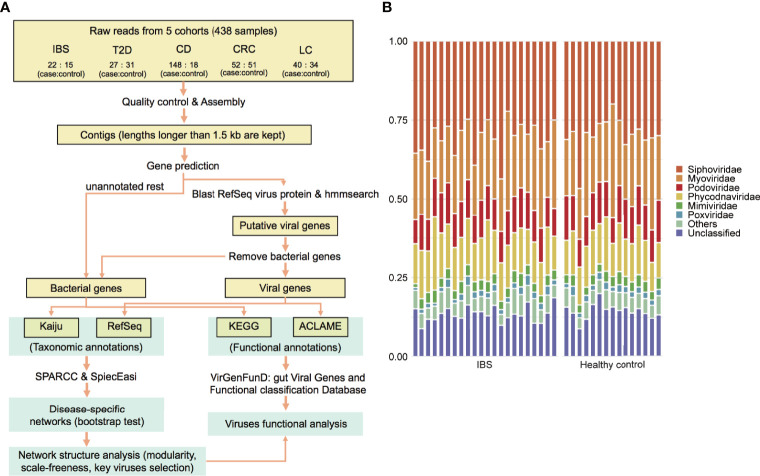
Viruses identified in the IBS and corresponding healthy controls. **(A)** Workflow of the analysis in this paper. **(B)** Viral composition in family level of each sample.

The overall analysis pipeline is shown in **Figure 1A**. After quality control and removing host sequences that mapped to the human genome, clean reads’ file size ranged from 2 to 28 GB. The metagenomic reads were then assembled into contigs by metaSPAdes (contigs longer than 1.5 kb were kept), and genes were predicted from these contigs by MetaGeneMark (Zhu et al., 2010; Nurk et al., 2017). We assumed that gene abundance was approximately proportional to the actual organism abundance. Thus, these genes were used to estimate the relative abundance of taxon among viruses and bacteria, respectively. For viral taxonomic annotation, genes were first aligned against the NCBI RefSeq non-redundant protein database (O'Leary et al., 2016) and then to the hidden Markov models of viral protein families generated from the JGI Earth’s virome project (Paez-Espino et al., 2016), in which 167,042 protein-coding genes from 2,353 isolated viral genomes were clustered into 14,296 protein families. To remove false positives that originated from bacteria, we removed genes that had significant hits to human gut bacteria contigs published in Forster’s study (Forster et al., 2019) (blastn, with arguments: -perc_identity 98, -qcov_hsp_perc 90, -evalue 1e-10, -max_target_seqs 1). The remaining union of genes that had hits against reference viral proteins and viral protein families were considered viral genes. We benchmarked the viral identification process by a simulated dataset, which revealed a specificity (true positive rate in the predicted viral genes) of 98.8% and a recall rate (true positive rate in all viral genes) of 54.0% (see details of benchmark in **Supplementary Methods**). Meanwhile, the non-viral genes were annotated with Kaiju (Menzel et al., 2016), which maps sequences to the NCBI RefSeq protein database containing bacteria and archaea proteins.

### Taxonomic Abundance Profile Calculation

All our taxonomic classifications were based on predicted genes. We first evaluated the contig abundances by mapping reads to contigs and calculated reads per kilobase: $a_{i}=\frac{x_{i}}{L_{i}}\times 1000$ , where *a_i_* is the abundance of contig *i* in sample *S*, *x_i_* is the number of reads mapped to contig *i*, and *L_i_* is the length of contig *i*. The gene abundance was calculated after adjusting the corresponding contig abundance by gene number in that contig: $g_{ij}=\frac{a_{i}}{n_{i}}$ , where *n_i_* is the number of genes in contig i. The abundance of a taxonomic unit was then added by the abundance of genes with the same taxonomic annotations: *t* = Σ g*_i j_*, $t'=\frac{t}{\sum t}$ , where *t*′ is the relative abundance of a taxonomy unit in sample S.

### Disease-Specific Co-Occurrence Network Construction

Based on the abundance matrix constructed by calculating the abundance of each feature, we used SparCC to measure the correlation between each pair of features (Friedman and Alm, 2012). One of the major factors that we need to take into consideration when inferring co-occurrence relationships of the microbiome is composition bias, which refers to the bias caused by the relative abundances that sum to 1 so that fractions tend to be negatively correlated regardless of their true relationships. SparCC is designed to estimate correlations of compositional data by the log transformation of pairwise data. Since SparCC adds a small value to each zero value to perform log transformation, correlations for elements that appear only in a few samples may not be reliable, so we removed elements that appear in less than 20% of the sample within a group.

After constructing networks of each case and control group by SparCC, the differential interaction between case and control groups of a cohort was divided into two parts: links that existed only in the network of the case group and links that existed in both networks but had significantly different correlation coefficients [*r*(*s_j_*,*s_j_* ′)]. To measure the significance of the difference of *r*, we calculated the difference (*D*, absolute value) of r between the same links that existed both in the network of case and control and compared it with the bootstrapped difference of r between cases and 100 shuffled co-abundance matrixes generated in SparCC. If *D* was beyond the 95% quantile of the bootstrapped difference, the interaction represented by this link was significantly different between cases and controls. The case-specific networks selected in this way were further filtered with SpiecEasi to get more reliable links and reduce indirect interactions (Kurtz et al., 2015).

### Pan- (Pooled) and Core- (Shared) Network Construction

We inferred the pan- and core-networks at the family level, since the pool of the genus-level networks had too many links to be visualized. The pan- networks were constructed by the pool of the links of the five disease-specific networks. The core network was constructed by the links that appear in four or more of the networks. We mapped the nodes in each network to the family level and then calculated the pan and core of the links. Links with the same source nodes and target nodes and the interaction type (positive or negative) were merged. Links with the same source and target nodes but different interaction types were removed.

### Network Topological Feature Computation

To quantify the characteristics of networks, we calculated node attributes such as degree, importance centrality, betweenness centrality, and network attributes such as average path length, modularity index, and scale-free index. The degree, importance centrality, and scale-free index were calculated with an in-house R script with the following method.

We denote *d(s_j_)* as the node degree, defined as the total edges that connect to the node *s_j_*:


$$d\left(s_{j}\right)=\sum_{\begin{array}{l} j'=1\\ j'\neq j \end{array}}^{N}\delta \left(s_{j,}s_{j'}\right)$$

We defined the node importance score (importance centrality) to denote the importance of a node by modifying the clustering coefficient (CC). Therefore, the importance score is defined as:


$$c\left(s_{j}\right)=\frac{d\left(s_{j}\right)\left(d\left(s_{j}\right)-1\right)+1}{2a\left(s_{j}\right)+1}$$

*a*(*s_j_*) is the number of edges that exist among the neighbor nodes that directly link to the node *s_j_*, not including *s_j_* itself. *b*(*s_j_*) is the maximum number of possible edges among the neighbor nodes of *s_j_*. This definition of CC is as the following:


$$\varphi \left(s_{j}\right)=\frac{a\left(s_{j}\right)}{b\left(s_{j}\right)}=\frac{2a\left(s_{j}\right)}{d\left(s_{j}\right)\left(d\left(s_{j}\right)-1\right)}$$

The reason to modify CC is that the numerator and denominator may be both 0 in *φ*(*s_j_*). Moreover, we want CC to be directly proportional to the importance of a node. That is, the more neighbor nodes of *s_j_* and fewer edges among these neighbor nodes, which means the more important of node *s_j_*, the larger the *c*(*s_j_*).

Betweenness: the node betweenness is defined by the number of shortest paths going through the node. It is calculated by the *betweenness* function of the “igraph” package in R (Csardi and Nepusz, 2006).

Scale-free index: if a network has the property where the low-degree nodes are in the majority and hub nodes of high degree are in the minority, the network is defined to be scale-free. The distribution of node degree in a scale-free network follows a power-law distribution, that is,


$$P\left(d\right)∼d^{-Y}$$

Thus, the scale-free index is defined as the fitness (*r*-square) of the linear regression model between log(*P*(*d*)) and log(*d*).

Modularity index: the modularity is calculated by *cluster_walktrap* and *modularity* function of the “igraph” package in R (Csardi and Nepusz, 2006), which utilize the random walk algorithm to cluster nodes in the networks.

Average path length: the average path length of the network is calculated by the *mean_distance* function of the “igraph” package in R.

### Viral Gene Functional Annotation and VirGenFunD Database Construction

We first annotated all genes of the microbiome with the KEGG database and the ALCME database separately (Leplae et al., 2010; Wang et al., 2015; Kanehisa et al., 2016). Since the KEGG functional annotation rate for viral genes was low (overall annotation rate: 46.8%), we used annotation of ALCME in later analyses (overall annotation rate: 80.1%). However, the majority of families in ACLAME were without GO or MeGO annotations, and thus we manually annotated them with the protein names that appeared most times within that family. After aggregating the families with the same annotation terms, a total of 2,162 function items were obtained. Thus, we further grouped these items into 16 categories manually by reference to COG (Tatusov et al., 2000). The details of the classification of the 16 categories are described in **Supplementary Methods**. We further built a database for the detected viral genes with manually classified functional annotation named VirGenFunD. Each sequence was labeled with annotations of taxonomy, KEGG, ACLAME, and VirGenFunD category and classified into five classes.

### Viral Gene Function Enrichment Analysis

The enrichment analysis was based on Fisher’s exact test. Here, we use all genes of viruses that take part in a network as the background. Since genes were both annotated with ACLAME family information and taxonomic information, the function of the VirGenFunD category can be retrieved through taxonomic annotation of genes. We first counted the number of each VirGenFunD category in the background list of viruses and then tested each VirGenFunD category for their enrichment in a subset of the background list by counting the number of a VirGenFunD category in that subset and performing Fisher’s exact test with a contingency table (take Category01 as an example):

**Table:** 

	Subset of background	Background
**Number of genes in Category01**	N_1_	N_2_
**Number of genes not in Category01**	N_3_	N_4_

### Statistical Information

Numbers that follow the ± sign in the manuscript indicate standard deviations. The Mann–Whitney U test was conducted with the *wilcox.test* function in R (two-sided), with a significance level set as *p* ≤ 0.05 for viruses and bacteria. The Kolmogorov–Smirnov test was used to test the normality of the distribution for continuous variables. The *t*-test was conducted with the *t.test* function in R (two-sided) for normally distributed continuous variables, with the significance level set as *p* ≤ 0.05 for metabolites. Fisher’s exact test was conducted with the *fisher.test* function in R. The networks constructed by SparCC were filtered with r ≥ *R*, and FDR-adjusted *p* ≤ 0.05, where r is the SparCC correlation coefficient; *R* was set as 0.6 in the IBS dataset and 0.4 in the other four datasets. The cutoff is different for the IBS dataset because the sample size is smaller than the other datasets. The network constructed is sensitive to sample size, and to make the network constructed more reliable and get the network of comparable size from different datasets, we set a higher cutoff for the IBS dataset. Statistics for disease-specific network selection were described above.

We used the permutation test to check whether negative or positive correlations were enriched in the relationships between viruses and bacterial phyla. For a given network, we first calculated the number of all bacteria–virus links (n1) and the number of negative bacteria–virus links (n2). For a given bacterial phylum (for example, *Firmicutes*), we calculated the number of negative *Firmicutes*–virus links (n3). We sampled n2 links from all the bacteria–virus links (n1) and calculated the number of *Firmicutes*–virus links (n4) 100,000 times. *p-*value was then calculated as the upper or lower quantile of n3 in the bootstrapped set of n4. The significance level was set as FDR *p* ≤ 0.05.

To test the significance of network structures (modularity, scale-free index, and average path length), we built networks of the null models through randomization of preserved number of nodes and edges. The *p*-values were calculated based on the 1,000 randomized null networks.

The major codes of our analyses are available at https://github.com/lkyvirrrr2001/viruses_analyses/.

## Results

### Consistent Variation of Viruses Along With Shifts of Bacteria and Metabolites in IBS

The alterations in gut bacteria between IBS and healthy controls have been reported in our previous work (Liu et al., 2016; Wang et al., 2019; Xu et al., 2020). Herein, we further explore the roles of the viral elements using these sequenced metagenomic samples, including 22 IBS patients (diarrhea-predominant) and 15 healthy controls, and 18 paired host metabolomic samples. The complete analysis pipeline is shown in **Figure 1A** and described in *Materials and Methods*. Our results demonstrated that the overall ratio of viral gene abundance to all the genes was 9.6% (±3.6%), with no significant difference between cases and controls. A total of 291 viral genera and 50 viral families were detected. Among the annotated viral families, *Siphoviridae*, *Myoviridae*, and *Podoviridae*, all belonging to the *Caudovirales* order, were the most abundant phages (**Figure 1B**), which are consistent with the typical composition of the phageome of human adults (Minot et al., 2011; Manrique et al., 2016; Ma et al., 2018). For eukaryotic viruses, *Megavirales*, *Pithoviridae*, *Baculoviridae*, *Nudiviridae*, *Nimaviridae*, *Circoviridae*, *Retroviridae*, *Togaviridae*, *etc*., were detected but were in the minor part. These viruses were also common in the human gut (**Table S1**) (Mukhopadhya et al., 2019).

**Figure 2:**
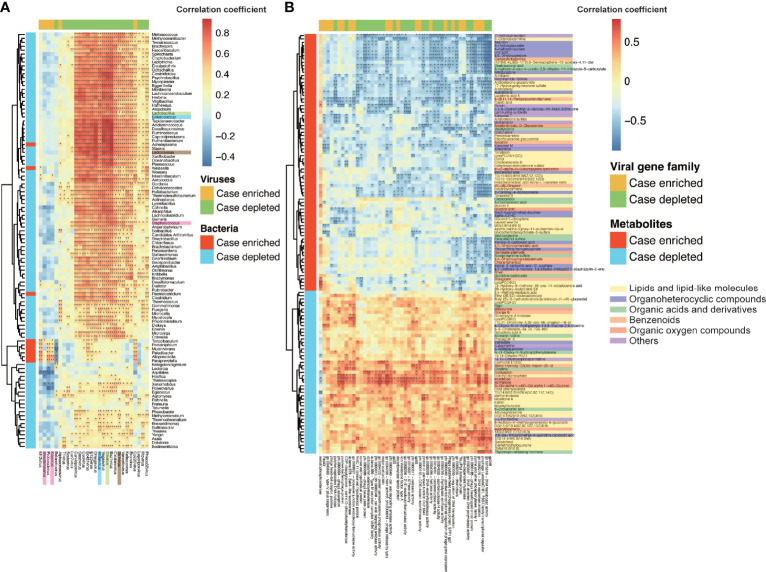
Correlations among viruses, bacteria, and metabolites. **(A)** Heatmap of the Spearman correlations between significantly different viruses (column) and significantly different bacteria (row). There were 22 viruses in genus level depleted and six enriched in the IBS group (Mann–Whitney U test, *p* ≤ 0.05), 97 bacteria in genus level depleted, and nine enriched in the IBS group (Mann–Whitney U test, *p* ≤ 0.05). The significances of correlations were labeled with “*” (FDR *p* < 0.05) and “**” (FDR *p* < 0.01). The same color-labeled names of bacteria or viruses indicate the pairs of infective phages and their bacteria hosts. **(B)** Heatmap of the Spearman correlations between significantly different viral gene families (column) and significantly different metabolic ions (row). There were 51 metabolic ions depleted and 85 enriched in the IBS group (*t-*test, *p* ≤ 0.05). The significances of correlations were labelled with “*” (FDR *p* < 0.05) and “**” (FDR *p* < 0.01).

The comparison between cases and controls revealed 22 and 6 viral genera depleted and enriched respectively in the IBS group (Mann–Whitney U test, *p* ≤ 0.05), detected by MetaComp (Zhai et al., 2017). In the previous study, the gut bacteria and host serum metabolites were found to have shifted between IBS and healthy controls (Xu et al., 2020). To explore whether these different viruses were related to the shifts of bacteria and metabolome between IBS and healthy controls, we calculated the Spearman correlations between different viruses and different bacteria (Mann–Whitney U test, *p* ≤ 0.05) as well as metabolites (*t-*test, *p* ≤ 0.05), respectively (**Figures 2A, B****)**. IBS-depleted viruses positively correlated with most of the IBS-depleted bacteria, and IBS-enriched viruses positively correlated with IBS-enriched bacteria (**Figure 2A**), showing a covarying relationship between shifted viruses and bacteria. Among the shifted bacteria, three lactic acid bacteria, *Lactobacillus*, *Lactococcus*, and *Enterococcus*, which are probiotics and beneficial to human health (Hatti-Kaul et al., 2018), were depleted in the IBS group (Mann–Whitney U test, *p* ≤ 0.05, **Table S2**). Alongside this shift, *C5virus* (phage that infects *Lactobacillus*), *Sk1virus* (phage that infects *Lactococcus*), and *Phifelvirus* (phage that infects *Enterococcus*) were also depleted in the IBS group (Mann–Whitney U test, *p* ≤ 0.05) and showed a significant positive correlation with their host bacteria (Spearman correlation, FDR *p* < 0.01), indicating a lysogenic relationship between the phages and their bacteria hosts.

The host non-targeted metabolomics profiling further supported the relationship between viruses and the disease. A total of 77 negative and 59 positive metabolic ions were detected as significantly different between cases and controls (*t-*test, *p* ≤ 0.05). These metabolic ions mostly came from lipids, amino acids, dipeptides, organic acids, disaccharides, benzenoids, *etc*. (**Table S3**), which can significantly separate samples of cases and controls by a partial least square discriminant analysis (PLS‐DA) model (**Figure S1**). Consistency between viruses and metabolome was observed herein, for IBS-depleted viruses positively correlated with IBS-depleted metabolic ions and negatively correlated with IBS-enriched metabolic ions (**Figure S2**). We also calculated the correlations between viral genes (annotated by ACLAME database) and the different metabolites, and some viral functions significantly correlated with the shifted metabolites (**Figure 2B**). Some of the correlated viral gene functions deal with viral propagation, such as replication, transcription, and transposition of related functions and structural proteins; others deal with enzymes that are important in biological functions for viruses or their host, such as virally encoded metal-dependent hydrolase, which catalyzes the hydrolysis of a wide range of biologically important substrates including carbohydrates, peptides, and nucleotides. Furthermore, the differential metabolites were involved in 47 metabolic pathways (**Table S3**), in which 25 overlapped with pathways found in the metagenomic data, and the differential viruses had a significant contribution to these pathways (11 pathways out of 25, Fisher’s exact test, *p* = 1.67 × 10^-2^). These results indicated that the changes of viruses accompanied changes in bacteria and metabolites of the IBS group, and the genes encoded by the viruses may affect the metabolic pathways related to human health.

### Characterization of the Gut Virome in Multi-Diseases

**Figure 3:**
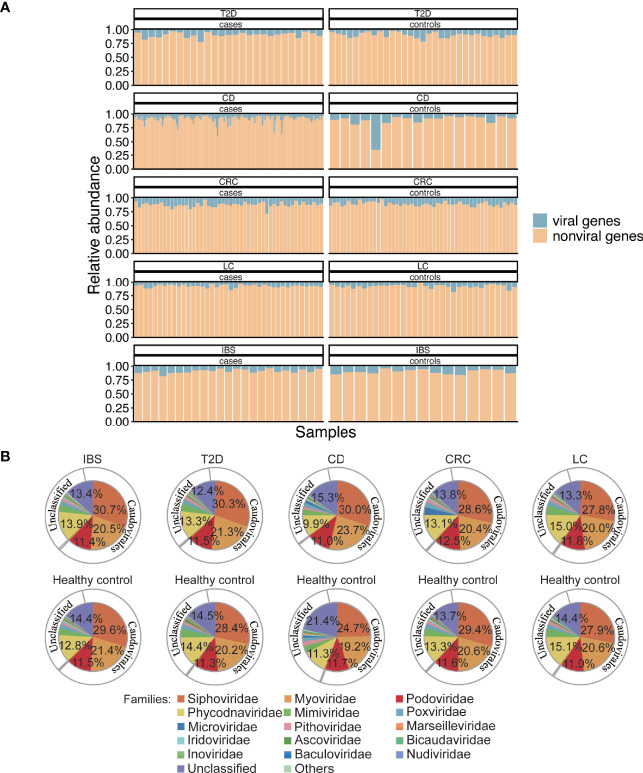
Abundance and composition of the gut virome in different groups. **(A)** Viral gene abundance in different samples. The blue part of each bar is the viral gene abundance relative to all the predicted genes in that sample and represents the portion of reads from the detected viral genes to reads from all genes in a sample. **(B)** Composition of viruses in family and order level. Inner pie chart, viral families. Outer circle, corresponding orders to the families. *Microviridae*, *Inoviridae*, *Bicaudaviridae*, and families that belong to *Caudovirales* are bacteriophages. *Phycodnaviridae*, *Mimiviridae*, *Poxviridae*, *Marseilleviridae*, *Iridoviridae*, and *Ascoviridae* are *Megavirales* known as nucleocytoplasmic large DNA viruses (eukaryotic viruses). *Pithoviridae*, *Baculoviridae*, and *Nudiviridae* are also eukaryotic viruses.

To get a better understanding of the gut virome in health and diseases, we collected metagenomic datasets from four other diseases, T2D, CD, CRC, and LC, which are typical metabolic or dysfunctional bowel diseases that are marked with dysbiosis in gut microbiota (Qin et al., 2012; Qin et al., 2014; Lewis et al., 2015; Yu et al., 2017). To explore the roles of viruses in a systematic view of diseases, we also collected published metagenomic datasets related to the above four diseases. Meta-data including age and sex of the involved individuals are listed in **Table S4**. All the raw data from the 438 gut metagenomic samples (including 37 samples in the above IBS cohorts) made up the total 5T size of fastq files which then followed a uniform analysis pipeline (**Figure 1A**). In the whole gene repertoire of the gut microbiome of all samples, viruses contributed 9.4% (±5.6%) of gene abundance to the total annotated genes (**Figure 3A**), and no significant difference in the viral ratio was found between each group of cases and controls. The compositions of viruses in the four datasets resembled that in the IBS dataset (**Figure 3B**), and identifiable phages occupy 68.5%–73.5% of all the viral abundance in different groups (**Figure S3**). In a systematic perspective of five diseases, there were 106 viral genera (**Table S5**) and 20 viral families (**Table S6**) that showed different abundances between cases and controls (Mann–Whitney U test, *p* ≤ 0.05). However, we found no shared different genus or family among the five datasets.

### Roles of Viruses in the Disease-Specific Co-Abundance Network of Gut Microbiota

To explore the possible positive or negative roles of viruses in the human gut with analysis of the disease-related microbiota samples herein, we thus investigated how phages and eukaryotic viruses respectively interacted with bacteria in disease-specific networks. We first constructed the co-abundance network of the microbes at the genus level within each case and control group (see bacterial and viral genus abundance matrixes in **Tables S2** and **S5**). Then, we selected links that specifically appeared in the case group as the disease-specific network (see *Materials and Methods*). We also constructed healthy-specific networks relative to each case group to make comparisons. Metrics to quantify the properties of the networks were calculated, including mean degree, edge number, importance centrality, betweenness, modularity, scale-free index, and average path length.

**Figure 4:**
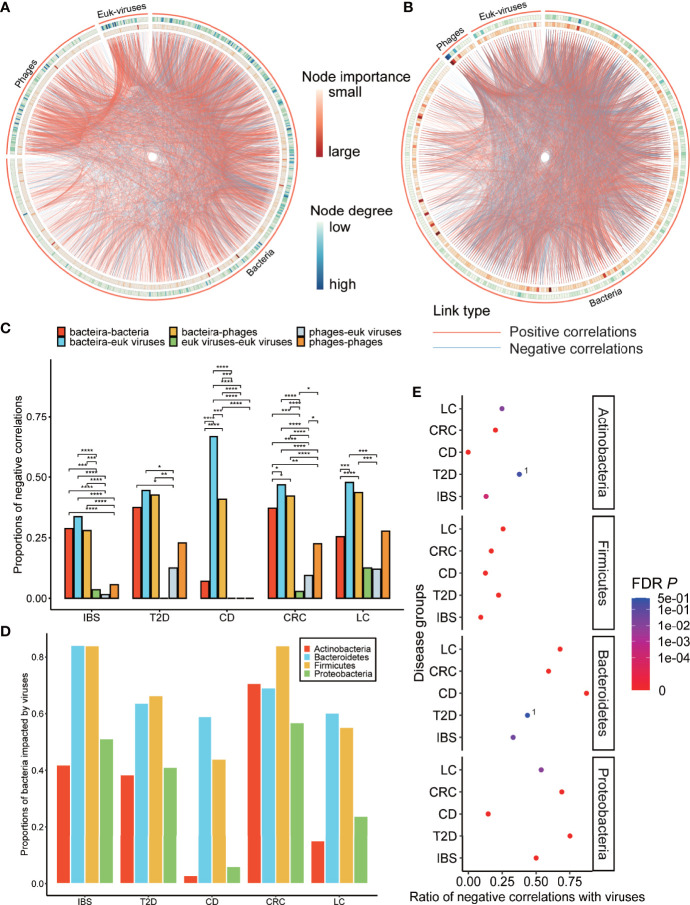
Characterization of disease-specific co-abundance relationships of viruses. **(A)** IBS-specific network. **(B)** Pan-network of five disease-specific networks in family level. **(A, B)** The co-abundance networks of the gut microbiome, including bacteria, phages, and eukaryotic viruses. The two ends of each edge represent two nodes (genus) that have interaction. Three types of annotations are outside the nodes: the outermost circles of the ribbon indicate three classes of the node; the two circles of the heatmap represent the node importance centrality and the node degree, which is defined as the number of nodes that link to each node. **(C)** Histogram of negative correlation ratios within and between three classes of nodes: bacteria, phages, and eukaryotic viruses in each disease-specific network (“*”; FDR *p* < 0.05, “**”; FDR *p* < 0.01, “***”; FDR *p* < 0.001, “****”; FDR *p* < 0.0001). **(D)** Proportion of four bacterial phyla that link to viruses. **(E)** Ratio of negative correlations of four bacterial phyla that link to viruses. Points with label “1” denote FDR *p* > 0.05. The *p*-values were calculated two-sided, so the negative correlation ratio close to 0 or 1 and FDR *p* < 0.05 means the positive correlation ratio or the negative correlation ratio (respectively) was enriched in the relationships between that bacteria and viruses. The negative link ratio in Proteobacteria and Bacteroidetes and the positive link ratio in Firmicutes and Actinobacteria were significantly higher than the random ratio (permutation test, FDR *p* is shown in the figure).

All the networks showed frequent correlations within and between bacteria, phages, and eukaryotic viruses, and some of the viral nodes were of high degree and high importance centrality (**Figure 4A**, **Figures S4****–** **S7**, and **Table S7**). The family-level pan (pooled) network and core (shared) network showed that phages, notably the three most abundant *Caudovirales* phages (*Siphoviridae*, *Myoviridae*, and *Podoviridae* families), took important positions in the network since they had both high degrees and high importance centralities (**Figure 4B** and **Figures S8****–** **S10**). The construction of the family-level pan (pooled) network and core (shared) network is described in *Materials and Methods*.

In all healthy-specific interaction networks, the mean degrees of viruses were comparable to those of bacteria. The mean degrees of eukaryotic viruses were even higher than those of bacteria in the healthy control group of the T2D dataset (**Table S8**, Mann–Whitney U test, W = 8079.5, *p* = 0.01), while in T2D, CD, and LC, the mean degrees of phages or eukaryotic viruses were smaller than those of bacteria which might indicate the decreased number of relationships of viruses with other microbes in these disease-specific networks (**Table S8**).

### Analysis of Relationships Between Viruses and Bacteria Suggested the Positive Role of Viruses

We then focused on the relationships between viruses and bacteria in disease-specific interaction networks to find how viruses impacted different bacteria phyla. Most of the relationships linked to viruses were from bacteria, indicating that viruses and bacteria had close and intricate interactions (**Figure 4A** and **Table S9**). Specifically, viruses, including both phages and eukaryotic viruses, had rarely negative relationships within themselves, compared to a high negative correlation ratio between viruses and bacteria (**Figure 4C** and **Table S9**, proportion test, FDR *p* < 0.05). This was also the case when we combined all the groups into a pan-group (**Figure S11**). The ratios of negative correlations in phage–phage correlation, phage–eukaryotic virus correlation, and eukaryotic virus–eukaryotic virus correlation (14.0%, 5.5%, and 3.1%, respectively) are significantly lower than the ratios of negative correlations in bacteria–phage correlation, bacteria–eukaryotic virus correlation, and bacteria–bacteria correlation (37.1%, 42.4%, and 25.9%, respectively) (proportion test, FDR *p* < 0.05). This result suggested that viruses might have restraints on some bacteria and rarely conflicted within themselves. *Firmicutes*, *Bacteroidetes*, *Proteobacteria*, and *Actinobacteria* were four major bacterial phyla that interacted with viruses, while *Firmicutes* and *Bacteroidetes* had the highest interaction ratio and thus might be the most impacted by viruses (**Figure 4D**).

Moreover, viruses showed different preferences in positive or negative correlations with these four bacteria. Viruses tended to have more positive correlations with *Firmicutes* and *Actinobacteria* and more negative correlations with *Proteobacteria* and *Bacteroidetes* (permutation test, **Figure 4E**), which are common dysbiosis-related and inflammation-mediating bacteria, respectively (Shin et al., 2015; Wassenaar and Zimmermann, 2018). This phenomenon was even more significant in healthy-specific networks (**Figure S12**), indicating a possible positive role of viruses to inhibit disease-mediating bacteria in both health and diseases.

### Viral Effects on Structures of Disease-Specific Networks

**Figure 5:**
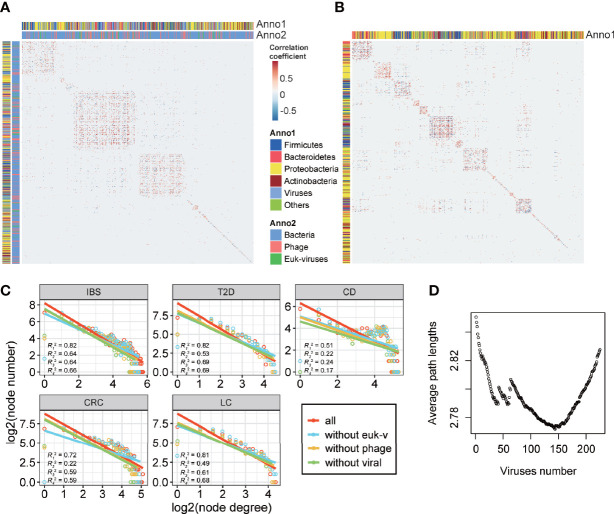
Structural indices of disease-specific networks. **(A)** Heatmap showing the modularity of the IBS-specific co-abundance network. **(B)** Heatmap showing the modularity of the IBS-specific co-abundance network after removing viral nodes. **(C)** Log2-transformed node degree distribution. Node degree is the number of direct links of a node. Degree distribution of a scale-free network follows a power-law distribution, and after log–log transformation, the distribution should fit a linear relationship. The r-square of each line is defined as the scale-free index. In each subgraph, R1 represents the line of all the nodes, R2 represents the line of nodes excluding eukaryotic viruses, R3 represents the line of nodes excluding phages, and R4 represents the line of nodes excluding all viruses. **(D)** Average path lengths of the pan-disease-specific network in family level. Viruses were added to the bacteria network in the decreasing order of betweenness centrality.

To further characterize the roles of viruses in the interaction networks of the disease-related microbiome, we calculated network structural features including modularity and scale-freeness of networks, to explore how viruses impacted the network structure. Modularity is a measurement of the property that a network can be divided into individual communities in which members are densely interconnected and sparsely connected outside. The modularity of biological networks makes the subgroups function semi-autonomously (https://psychology.wikia.org/wiki/Modularity_(biology)). In all healthy-specific and disease-specific networks, the modularity indices were significantly higher than those of random network null models with the same number of nodes and edges of each network (1,000 times of randomization, *p* < 0.001) (**Figure 5A** and **Figures S13****–** **S16**). These differences showed that relative independent communities existed among microbes, in which viruses interspersed among bacteria rather than grouped into individual modules. More interestingly, we explored the viral effects on the structures of networks by removing the virus nodes in disease-specific networks and observed that the modularity of the bacteria network increased in all groups (**Figure 5B** and **Table S9**), meaning that viruses connect between bacteria modules and contribute to shaping the network structure of the gut microbiota.

The scale-free property of networks, in which a few nodes possess a large number of relationships, and most nodes possess a small number of relationships, is typical of biological systems that are robust to random disruptions (Barabási and Albert, 1999). Herein we calculated scale-free indices, defined as the *r*-square of the node degree fitting a log-transformed power-law distribution, of the five disease-specific interaction networks (**Table S9**). All the networks showed the characteristics that low-degree nodes had higher frequencies than high-degree nodes (**Figure 5C**, 1,000 times of randomization, *p* < 0.001), and short paths existed among different taxa (**Table S9**), compared to the average path length of six in random networks (1,000 times of randomization, *p* < 0.001) (Watts and Strogatz, 1998; Faust et al., 2012). This result indicated that the disease-specific networks had the property of scale-freeness. When comparing the scale-free indices of the total networks with the ones in which viruses were not considered (non-viral), we found that the scale-free indices of all the networks decreased (**Table S9**, 1,000 times of randomization, *p* < 0.001). Although the decrease in scale-free indices was mainly attributed to the removal of substantial amounts of low-degree viral nodes, this result indicated that viruses improved the scale-freeness of the networks and made the microbial community of gut microbiota more robust.

The network structure analysis illustrated that viruses could bridge between bacteria, but not all viruses were equally important. To determine which viruses were more important in communicating between bacteria, we defined the ones that could shorten all the bacteria’s average path lengths as key viruses. By calculating the betweenness centrality of each viral node and adding them to the bacterial community in decreasing order, we found lists of such key viruses that minimize the average path length in the pan network as well as five disease-specific networks (**Figure 5D** and **Table S10**). These viruses took significant parts in interacting with short-chain fatty acid-producing bacteria *Firmicutes* (121 out of 125 interacting bacteria, Fisher’s exact test, *p* = 1.58 × 10^-2^) and *Bacteroidetes* (79 out of 83 interacting bacteria, Fisher’s exact test, *p* = 6.23 × 10^-2^), which implied that in the disease-specific networks, viruses might take an important part in the gut metabolism, by the coaction with these bacteria.

### Viral Gene Functional Annotation Revealed Their Roles in Disease-Related Microbial Community

**Figure 6:**
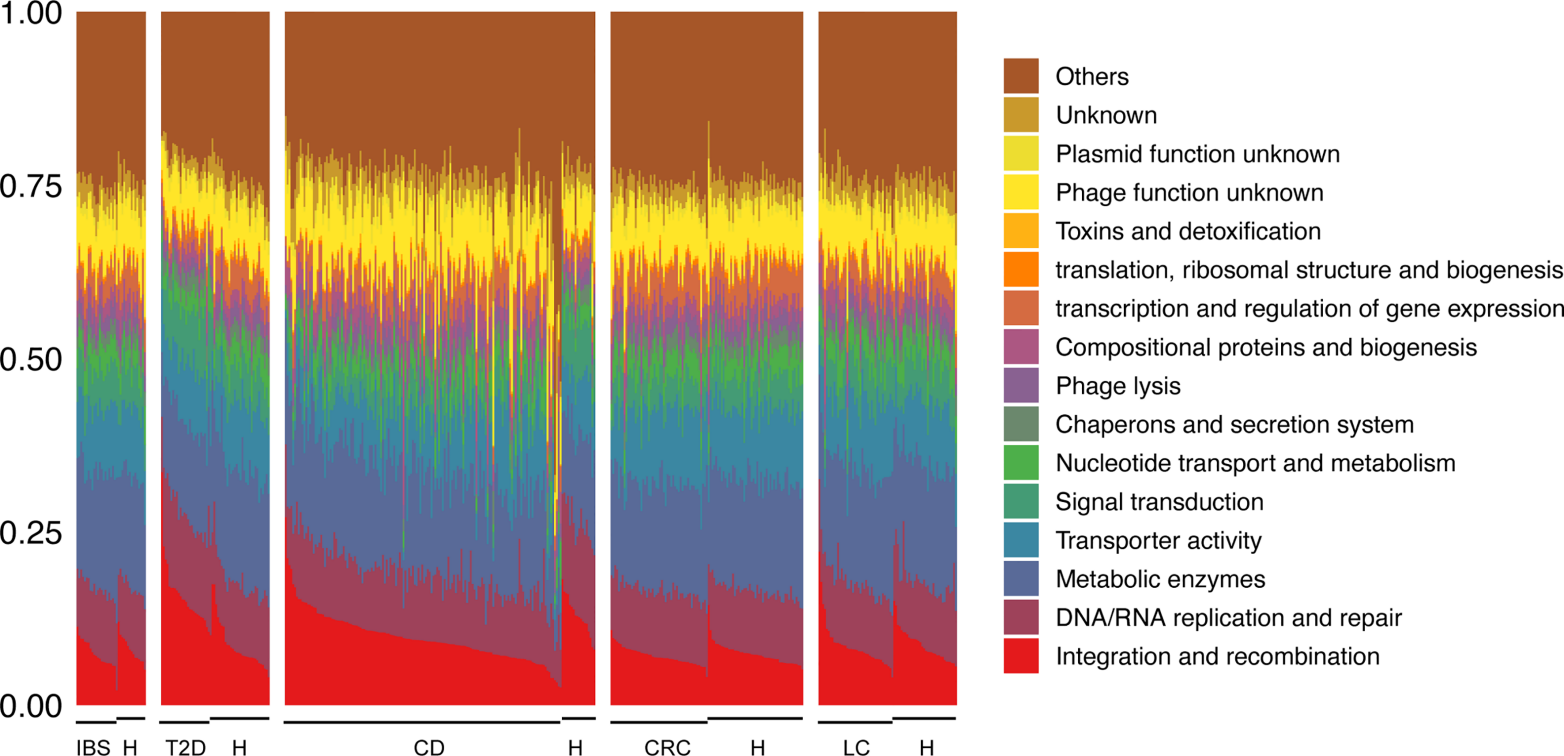
Composition of 16 categories of viral gene functions based on the VirGenFunD functional annotations. Samples from the same data source were grouped, and the label “H” stands for healthy controls. Not surprisingly, some conserved backbone functions such as phage integration and transpositional recombination, DNA/RNA replication, and repair occupied a substantial part of the whole viral functions. Phage lysis, metabolic enzymes, transporter activity, and signal transduction-related function were also active in viral gene functions. At the same time, accessory gene functions such as toxins and detoxification were also detected.

To further explore how viruses interact with bacteria, we first investigated the viral gene functions by annotating them with viral protein families in the ACLAME database. Since only a small part of protein families are annotated with GO or MeGo, we reannotated them and categorized them into 16 categories (**Figure 6** and **Table S11**). Based on the 16 categories, we performed enrichment tests on gene functions of viruses that interact with bacteria (see *Materials and Methods*). The gene set of key viruses selected in each network described above was generally enriched in functions of “Transporter activity”, which mainly includes ATP-binding cassette (ABC) transporter- and transmembrane transporter-related proteins, “Chaperons and secretion system”, which mainly includes proteins involved in type III and type IV secretion system that transport molecules from bacterial cells to other cells, “Metabolic enzymes”, which include general enzymes such as oxidoreductase, hydrolase, and modification-related enzymes, and “Signal transduction”, which mainly includes a two-component signal transduction system and response to stress (**Table S12**, Fisher’s exact test, FDR *p* < 0.01). These functions are essential biological functions for both viruses and hosts. Viruses in these ways might affect the phenotype of bacteria under the condition of diseases.

We further tested the significance of functional enrichment of phages that negatively and positively interacted with bacteria (phages that had negative link ratios of 1 and 0 were selected as representatives, respectively). In comparison with all the annotated phage genes, functions that were typical of temperate phages such as “Integration and recombination” and “Metabolic enzymes” were enriched in both positive and negative interacting phages with bacteria (**Tables S13** and **S14**, Fisher’s exact test, FDR *p* < 0.01), supporting that most phages in the gut were temperate phages (Toussaint and Rice, 2017). Function “Transcription and regulation of gene expression” and “Nucleotide transport and metabolism” were only enriched in phages that positively interact with bacteria. In contrast, function “Toxins and detoxification” (this category includes toxins, post-segregating killing process, and detoxification of mercury ion) was only enriched in phages that negatively interact with bacteria. These results indicated the possible mechanisms underlying the correlations between phages and bacteria, either through direct predation or lysogeny, or through indirect interaction between phages and non-host bacteria by affecting the host gene expression, metabolism, and virulence. We performed the same analysis in the healthy-specific networks but found no positive-interaction-specific or negative-interaction-specific viral function, indicating a homogeneous distribution of gene functions in positive and negative interacting phages with bacteria (**Tables S15** and **S16**). As for eukaryotic viruses, which were generally ignored in the gut virome, they interacted with bacteria with the enriched functions of “Signal transduction”, “Metabolic enzymes”, and “Transporter activity” (**Tables S17** and **S18**, Fisher’s exact test, FDR *p* < 0.01) in both disease- and healthy-specific networks, giving hints to the potential roles of eukaryotic viruses in the gut.

To provide convenience for further analyses and more insights into the role of viruses in the human gut, we summarized our detected viral sequences and annotations from different sources into a database named VirGenFunD. Although there are some excellent viral databases like GVD (Gregory et al., 2020), ACLAME (Leplae et al., 2004), and pVOGs (Grazziotin et al., 2016), which include large amounts of viral sequences, they either lack functional annotations or have sporadic GO annotations. The VirGenFunD database contained 3,351,765 viral gene sequences from five disease-related datasets and the corresponding healthy controls. The VirGenFunD annotation thus nearly doubled the number of the known function categories (**Table 1**) and provided vital clues for understanding the functions of viruses in the human gut. The VirGenFunD database is available at http://cqb.pku.edu.cn/ZhuLab/VirGenFunD/, https://yjiang724.github.io/VirGenFunD/.

**Table 1:** Comparison of the number of annotations for viral sequences in VirGenFunD.

	Total viral genes (number)	Taxonomic annotation	KEGG	VirGenFunD	VirGenFunD increased annotations
IBS	583,659	516,827	276,611	470,880	220,037
T2D	362,294	313,191	166,848	284,793	134,589
CD	671,020	583,808	292,984	530,029	264,582
CRC	1,489,429	1,325,241	722,396	1,205,490	552,547
LC	245,363	210,944	110,729	192,309	92,851
Total	3,351,765	2,950,011	1,569,568	2,683,501	1,264,606

## Discussion

In this study, we emphasized the importance of the roles of viruses in the human disease-related gut microbiome. As revealed in previous studies, gut viruses play important roles in maintaining the healthy human gut (Minot et al., 2011; Manrique et al., 2016). Here, we focused on how viruses shaped the microbial community in the disease-related gut. Our results suggested that viral genes accounted for substantial amounts of genes in the gut microbiome, yet we suspected that the amounts of viruses were underestimated under our relatively strict viral gene identification standard. Eukaryotic viruses, whose roles were rarely studied, also had non-negligible amounts in the gut microbial community. The phages detected in our study mainly were *Caudovirales*. Noticeably, *Microviridae*, reported to be one of the dominant phages in some studies (Norman et al., 2015; Shkoporov et al., 2019), were not very abundant in our results. This could be caused by the missing of free ssDNA viruses in the library preparation of metagenomic sequencing and that *Microviridae* might be overestimated in some studies due to the bias to ssDNA viruses during the multiple displacement amplification of enriched viral DNA (Kim and Bae, 2011). We also observed that lists of viruses showed shifted abundance between cases and controls in one or more diseases of our five datasets. These viruses might relate to the metabolite changes of the human host, which could be supported by the metabolomics data of the IBS group and the reported diversity correlation between phages and bacteria (Moreno-Gallego et al., 2019).

Our result showed that the majority of shifted gut bacteria covaried with shifted viruses, which goes with Coughlan’s study that found virome alterations partially covaried with bacteriome alterations (Coughlan et al., 2021). Especially, the positive correlation between the lactic acid bacteria (*Lactobacillus*, *Lactococcus*, and *Enterococcus*) depleted in IBS and their phages indicate the mutualistic relationship between phages and these probiotics, showing a positive role of viruses as well. It is worth studying whether these probiotics can have better colonization together with their phages as intake.

The metabolomic analysis of IBS showed that some metabolites of lipids and amino acids changed in the IBS group. The changes of metabolites could be related to the pathogenesis of IBS, including gastrointestinal motility, visceral sensation, intestinal permeability, and gut microbiota which are among the key factors affecting the pathogenesis of IBS (Chey et al., 2015; Pimentel and Lembo, 2020). These correlations observed among viruses, bacteria, and metabolites indicated a cofactor role of viruses together with bacteria in IBS. Moreover, shifted viral gene functions significantly correlated with the shifted serum metabolites. These viral gene functions included transcriptional regulators, chemotaxis, and enzymes, through which viruses can interact with bacteria. These results suggested that viruses might affect the gut physiology by indirectly modulating phenotypes of hosts or producing metabolic enzymes that can affect the metabolome of the gut.

In the analyses of gut viruses of multiple diseases, although batch effects should exist in datasets of different studies’ interaction networks, they do not affect the later analysis since there is no difference comparison between different studies. In the analyses of co-occurrence networks, we found a complex interplay between viruses and bacteria, and some viruses were in the hub position of the disease-specific networks. These results supported that viruses, which included both phages and eukaryotic viruses, were not silent in the gut but were actively interacting with each other and bacteria. Phages have natural parasitic relationships with bacteria, and it is not surprising that phages are affected by bacteria or affect bacteria in the community, through either the lysogenic mutualism or the proactive lysis with their host bacteria. On the other hand, eukaryotic viruses also showed substantial relationships with bacteria, giving us hints that the relationship between eukaryotic viruses and bacteria might be important in the microbial community and deserves more attention.

The current study showed a preference for viruses to more negatively correlate with *Proteobacteria* and *Bacteroidetes* in both healthy and diseased samples. *Proteobacteria* are usually the signature of dysbiosis in gut microbiota (Shin et al., 2015), and *Bacteroidetes* are lipopolysaccharides-producing and inflammation-mediating organisms whose overabundance is associated with diseases such as liver damage, chronic inflammation of the gut, and diabetes (Wassenaar and Zimmermann, 2018). Since the abovementioned five diseases all deal with aberrant immune responses and varying degrees of inflammation (Brenner et al., 2015; Brennan and Garrett, 2016; Zuo and Ng, 2018; Sharma and Tripathi, 2019; Pimentel and Lembo, 2020), this result showed a possible ameliorating effect of viruses on the disease-related gut. We deemed this hypothesis highly possible since there is evidence that phages can protect the mammalian hosts from harmful bacteria (Barr et al., 2013; Barr et al., 2015). Besides, phages also have recently been used as brief clinical antimicrobial agents, but their intake as treatment for inflammation or microbiota dysbiosis is faced with huge challenges since phages may have a complex effect on the microbial ecology (Febvre et al., 2019). Our results suggested that phages that may reduce disease-unfavorable bacteria exist naturally in the microbiota-disordered gut and may offer helpful insights to the designing of supplemental phage drugs.

The network structure analysis suggested that viruses bridged between bacteria and contributed to the robustness of the networks. The modularity of networks can represent core and peripheral specialized metabolic functions of the microbiome (Vitkup et al., 2006; Kreimer et al., 2008), and our results showed that the key viruses of high betweenness centrality took significant parts in interacting with nearly all *Firmicutes* and *Bacteroidetes*, which are the major source of producing short-chain fatty acids the key viruses interacted with. This result indicates that viruses may take part in the metabolic networks of the gut. Besides, the functional enrichment of the key viruses also suggested the important role of prophages in metabolism and communication with bacteria through expressing genes that may modulate the phenotype of hosts, such as transcriptional regulators, toxins, and enzymes.

The functional enrichment analysis enabled us to view through what functions the viruses interact with bacteria. Results showed that viruses interact with bacteria through predation (suggested by the function of phage lysis), expressing genes involved in the transporter and secretion system, metabolic enzymes, *etc.* However, as has been suggested in the altered network analyses and addressed in many other studies that metabolic functions or pathways shifted in diverse diseases (Greenblum et al., 2012; Heinken et al., 2021), the specific positive or negative role of viruses on their involvement in metabolism and on their functional modification to their hosts in diseases still needs further exploration.

For eukaryotic viruses, more and more studies have shown that they can interplay with bacteria in various scenarios. For example, bacteria help enhance virion stability and affect attachment and infectivity for some viral pathogens to viral hosts, and bacteria can regulate the intestine immunity to viruses (Berger and Mainou, 2018). While the mechanism of how eukaryotic viruses interact with bacteria is still obscure, our results demonstrated that these viruses were enriched in some key functions such as “Signal transduction,” “Metabolic enzymes,” and “Transporter activity.”

Several limitations to our study herein should be pointed out. First, the viral genes identified in our pipeline are more reliable than sensitive, which may lead to the missing of some low-abundance viruses and thus obscuring of some variations between individuals. However, we believe our conclusions will not be fluttered by the missed viruses, which the aggregation of the five diseases can compensate. Second, the identification of viral genes was largely based on the reference database, so unknown viruses were not included in part of our analysis. Therefore, we used genes to evaluate the taxonomic composition at least at the viral genus level, which can elevate the annotation rate through homologous genes at the species or strain level. In fact, there are several excellent viral gene detection tools which can be used in future analyses (Fang et al., 2019; Fang et al., 2020). Third, some samples in the CD case group were treated with antibiotics. This dataset may not be able to perfectly reflect the bacteria composition of CD, but we can still infer the roles of viruses by the relationship with bacteria. Lastly, some results in our study (for example, differential viruses) may be inconsistent with similar studies that utilize the viral-like particle (VLP) enrichment virome sequencing method. That is because the VLP enrichment method only sequences free viral particles, without considering prophages. Both strategies are important in virome studies and should be chosen based on different goals of studies. Thus, the comparisons between our results and these studies can be difficult.

Altogether, this study provided a landscape of the roles of viruses in the disease-related gut microbiome. Gut viruses altered between diseases and controls together with the bacteria. Although the causative relationship between the change of viruses and bacteria cannot be determined, positive roles of viruses have been suggested by our results. For example, phages of beneficial bacteria that produce lactic acids covaried with their bacteria hosts, both of whose abundances are depleted in the IBS group. Besides, viruses showed the potential to inhibit the unfavorable bacteria in the disease-related gut, thus maintaining the relative wellbeing of the gut functions. Key viruses screened in the interaction networks showed their indispensable role in gut metabolism. The functional analyses of viral genes also provide vital clues for understanding the mechanisms of the interactions between viruses and bacteria. Our study can provide a better understanding of the gut microbial community and may offer new insights into the future treatment-related studies of different diseases.

## Data Availability Statement

The datasets presented in this study can be found in online repositories. The names of the repositories and accession numbers can be found as follows: The IBS dataset is deposited in the European Nucleotide Archive with accession number PRJEB40628 (https://www.ebi.ac.uk/ena/browser/view/PRJEB40628). The T2D dataset is deposited in the NCBI Sequence Read Archive with accession numbers SRA045646 (https://www.ncbi.nlm.nih.gov/sra/?term=SRA045646) and SRA050230 (https://www.ncbi.nlm.nih.gov/sra/?term=SRA050230). The CD dataset is deposited in the NCBI Sequence Read Archive with accession number SRP057027 (https://www.ncbi.nlm.nih.gov/sra/?term=SRP057027). The CRC dataset is deposited in the European Nucleotide Archive with accession number PRJEB10878 (https://www.ebi.ac.uk/ena/browser/view/PRJEB10878). The LC dataset is deposited in the European Nucleotide Archive with accession number PRJEB6337 (https://www.ebi.ac.uk/ena/browser/view/PRJEB6337).

## Ethics Statement

The studies involving human participants were reviewed and approved by the Ethics Committee of Peking University People’s Hospital (No. 2017PHB105-01). The patients/participants provided their written informed consent to participate in this study. Written informed consent was obtained from the individual(s) for the publication of any potentially identifiable images or data included in this article.

## Author Contributions

HZ and XJ co-supervised the study. HZ, ML, and XJ designed the study. ML performed the major analyses and database construction. CW collected the information of CRC source data and helped with data analysis. QG examined the manuscript and offered valuable suggestions. CX collected another three groups of source data except for CRC. ZX helped with the plotting. All authors contributed to the article and approved the submitted version.

## Funding

This work was supported by the National Key Research and Development Program of China (2021YFC2300300, 2017YFC1200205) and the National Natural Science Foundation of China (32070667, 31671366).

## Conflict of Interest

The authors declare that the research was conducted in the absence of any commercial or financial relationships that could be construed as a potential conflict of interest.

## Publisher’s Note

All claims expressed in this article are solely those of the authors and do not necessarily represent those of their affiliated organizations, or those of the publisher, the editors and the reviewers. Any product that may be evaluated in this article, or claim that may be made by its manufacturer, is not guaranteed or endorsed by the publisher.
