# Supplementary material for: More Positive or More Negative? Metagenomic Analysis Reveals Roles of Virome in Human Disease-Related Gut Microbiome
Source: Front Cell Infect Microbiol. 2022 Apr 12;12:846063. doi: 10.3389/fcimb.2022.846063 (PMC9040671; doi:10.3389/fcimb.2022.846063)
Supplement: Supplementary file 2 [file DataSheet_2.docx]

Supplementary Material

# Supplementary methods

**1.1 Enrollment of IBS patients and healthy controls**

The enrollment of all subjects is in cooperation with Duan Liping's research group from the Department of Gastroenterology, Peking University Third Hospital. We recruited a group of IBS patients aged 18-65 years with IBS-D and healthy individuals and collected the fecal samples. Among them, IBS patients meet the diagnostic criteria of Rome III. Healthy controls were additionally recruited healthy volunteers. Exclusion criteria: individuals with organic gastrointestinal disease such as IBD or diabetes; individuals with concurrent infections of the respiratory, digestive or urinary systems; individuals who have had abdominal surgery in addition to appendicitis; individuals who have used antibiotics or antidepressants in one month; individuals who have used probiotics or antidiarrheals for more than three days in two weeks; pregnant and breastfeeding women are excluded in this study. Each individual underwent a colonoscopy to ensure that they had no organic bowel disease. The final number of IBS patients who met the criteria was 22, and the number of healthy controls was 15.

**1.2 Sample collection and DNA extraction**

Fecal samples were stored in a -80°C freezer immediately after collection. Fecal microbial DNA was isolated using MagPure Stool DNA KF Kit B (Magen, China) according to the manufacturer's instructions. Samples were then analyzed using Qubit dsDNA BR Assay Kit (Invitrogen, USA) for fluorescence quantification and mass detection and then quality controlled with aliquoting 1% agarose gel. After DNA extraction, 1 µg of genomic DNA was randomly fragmented using a Covaris ultrasonicator and purified by the AxyPrep Mag kit. DNA fragments with an average length of 200-400 bp were screened by the Agencourt AMPure XP Medium kit. Next, DNA is end-repaired and purified by End Repair Mix. The repaired DNA was mixed with A Tailing Mix, followed by ligation of Illumina adapter sequences to the adenylate 3' end DNA, followed by further purification. Products were selected based on the length of the inserted sequences, and several rounds of PCR amplification were performed with PCR Primer Cocktail and PCR Master Mix. After purification, the libraries were checked by an Agilent 2100 Bioanalyzer (Agilent, USA) and ABI StepOnePlus real-time PCR system. Finally, qualified libraries were sequenced on the Illumina Hiseq platform (BGI, Shenzhen, China)

**1.3 Non-targeted metabolomic assay**

Serum metabolomic assays were performed on 18 of 37 subjects, including nine IBS patients and nine healthy controls. All serum samples were collected by an LC-MS system, and all chromatographic separations were performed using an ultra-high performance liquid chromatography system. First, reversed-phase separation was performed using an ACQUITY UPLC BEH C18 Column (100 mm x 2.1 mm, 1.7 μm, Waters), the oven was kept at 50 °C, the flow rate was 0.4 mL/min, and the mobile phase consisted of solvent A (water + 0.1 % formic acid) and solvent B (acetonitrile + 0.1% formic acid). The gradient elution conditions were set as follows: 0-2 min, phase A 100%; 2-11 min, phase B 0% to 100%; 11-13 min, phase B 100%; 13-15 min, phase A 0% to 100% 100%. The injection volume for each sample was 10 μL.

A high-resolution tandem mass spectrometer Xevo G2 XS QTOF (Waters) was used to detect metabolites eluted from the column, and the Q-TOF was run in positive and negative ion modes. For positive ion mode, capillary voltage and sampling cone voltage were set to 3 kV and 40 V, respectively. For negative ion mode, capillary voltage and sampling cone voltage were set to 1 kV and 40 V, respectively. Mass spectrometry data were acquired in centroid MSE mode with a TOF mass range of 50 to 1200 Da and a scan time of 0.2 s. For MS/MS detection, all precursors were cleaved using 20–40 eV with a scan time of 0.2 s. During acquisition, the LE signal was acquired every 3 s to calibrate the mass accuracy. To evaluate the stability of LC-MS throughout the acquisition process, a quality control sample was collected from all sample pools after every 10 samples.

**1.4 Screening of differential metabolites**

The metabolomic data were subjected to multivariate statistical analysis by partial least squares discriminant analysis (PLS-DA) model. To identify the major metabolites capable of discriminating between different sample groups, we used the variable importance (VIP) metric in the projection plot to indicate the contribution of each feature to the regression model. The higher the VIP score, the greater the contribution of the metabolites to distinguishing the different groups. In this paper, the VIP values ​​of the first two principal components of the PLS-DA model were combined with univariate analysis of fold change and *P*-value to screen for differential metabolites. The screening conditions are 1) VIP ≥ 1; 2) fold-change ≥ 1.2 or ≤ 0.8333; 3) *P* < 0.05, and the ions that satisfy these three conditions at the same time are selected as differential metabolic ions.

**1.5 Benchmark of the viral gene identification**

To evaluate the accuracy of our strategy to detect viral genes, we constructed a dataset of known composition and tested on it. The synthetic metagenome used for benchmark consisted of 613 human gut bacteria genomes, 1,726 phage genomes, two eukaryotic virus genomes, and three archaea genomes. All the genomes were first gene-predicted by MetageneAnnotator implemented in VirSorter^3^. Among the bacteria genomes, a total of 1,905,972 genes were predicted from bacteria genomes, among which 619,808 genes were detected to be putative phages or prophages by VirSorter in the categories 1, 2, 4, and 5, which were sure or somewhat sure to be true phage genes or prophages. These putative phages and prophages were removed from the true bacterial genes for the subsequent benchmark. And among the archaea genomes, no putative phages or prophages were detected. In total, 1,286,164 bacterial genes, 6,419 archaeal genes and 174,607 viral genes were used to test the accuracy of our pipeline. The result showed a specificity (true positive rate in the predicted viral genes) of 98.8% and a recall rate (true positive rate in all viral genes) of 54.0%.

**1.6 Functional and taxonomic abundance profile calculation**

Reads were mapped to contigs to evaluate the abundance of each contig with bowtie2 arguments –fast, --no-unal, --no-head. Abundance of contig i of sample S was calculated as number of reads of sample S mapped to this contig and then adjusted by the length of the contig, which was defined as reads per kilobase:

$$a_{i}= \frac{x_{i}}{L_{i}} \times1000$$

$a_{i}$: the abundance of contig i in sample S.

$x_{i}$: the number of reads mapped to contig i.

$L_{i}$: the length of contig i.

We assigned the abundance of contig i to each gene in that contig. So, the abundance of a unit in function k was calculated as:

$$g_{ij}=a_{i}$$

$$k=\sum g_{ij}$$

$$k^{'}=\frac{k}{\sum k}$$

$g_{ij}:$ the abundance of gene j in contig i.

$k:$ the abundance of a functional unit in sample S.

$k^{'}:$ the relative abundance of a functional unit in sample S.

In the calculation of taxonomic abundance calculation, we assume that a contig represents a species, hence the abundance of genes should be adjusted by the number of genes in each contig. Thus, the abundance of a taxonomic unit t is calculated as:

$$g_{ij}=\frac{a_{i}}{n_{i}}$$

$$t=\sum g_{ij}$$

$$t^{'}=\frac{t}{\sum t}$$

$g_{ij}:$ the abundance of gene j in contig i.

$n_{i}:$ the number of genes in contig i.

$t:$ the abundance of a taxonomy unit in sample S.

$t^{'}:$ the relative abundance of a taxonomy unit in sample S.

Profiles of higher levels in taxonomy were calculated based on the species profiles of each sample. Although there were situations where different species of genes were annotated on one contig, we thought this bias could make up by the neutralization of large amounts of genes. The taxonomy mapping was aided by the R package 'taxize'.

**1.7 Co-occurrence network construction**

The correlations of all networks were calculated in the same procedure. The script SparCC.py was first used to calculate the correlation coefficient between all features, with argument -i 5. *P*-values of each correlation were inferred from the method of bootstrap. First, 100 shuffled datasets were made using script MakeBootstraps.py. Then, correlation coefficients were calculated within each dataset by SparCC.py as the same argument as above. Finally, PseudoPvals.py was run to get the pseudo *p*-values with argument -t 'two_sided'. In an undirected network consisting of N nodes, the relationship between one node *s_j_*(*j* =1,2,...,*N*) and another node *s_j'_* (*j'* =1,2,...,*N*) was given by:

$$\delta\left( s_{j},s_{j^{'}} \right)=\left\{ \begin{aligned} 1, &if \left| r\left( s_{j},s_{j^{'}} \right) \right|\geq R\mathrm{and}q\left( s_{j},s_{j^{'}} \right)<0.05; \\ 0, &else， \end{aligned} \right.$$

*r* is the SparCC correlation coefficient, and *q* is the FDR adjusted *p*-value. R was set as 0.6 in IBS dataset, and 0.4 in other four datasets. If $\delta\left( s_{j},s_{j^{'}} \right)$ equals 1, an edge exists between *s_j_* and *s_j'_*.

**1.8 VirGenFunD classifications**

The classification of 16 functional categories in VirGenFunD database was based on the family annotation of ACLAM database. Since only a minority of the ACLAME families have annotations of GO or MeGO, we manually annotated the rest families without GO or MeGO with the protein names that appear the most times within that family. After aggregating the families with the same annotation terms, a total of 2,162 function terms were obtained. Thus, we further grouped these terms into 16 categories manually by reference to classification in COG database. Category01 is 'Integration and recombination', which mainly includes function of phage DNA integration, tyrosine-based recombinase activity, site-specific DNA recombination, DDE-based recombinase activity and transpositional DNA recombination. Category02 is 'DNA/RNA replication and repair', which includes proteins such as polymerase, helicase, topoisomerase, ligase activities and DNA mismatch repair proteins. Category03 is 'Metabolic enzymes'. This category is grouped because some generally described proteins cannot be classified into a specific function, for example, oxidoreductase activity, hydrolase activity, and modification-related activity. Category04 is 'Transporter activity', which includes ATP-binding cassette (ABC) transporter, transmembrane transporter, as well as some host-related pathways such as response to drugs, detoxification to mercury ion, response to antibiotics. Category05 is 'Signal transduction', which mainly includes two-component signal transduction system and proteins that response to stress. Category06 is 'Nucleotide transport and metabolism', which includes proteins involved in nucleotide biosynthesis and metabolism activities. Category07 is 'Chaperons and secretion system', which include all sort of chaperons and proteins in secretion system (mainly type III and type IV secretion system). Category08 is 'Phage lysis', which includes functions related to endolysin activity, phage release by lysis activity and cell wall degrading amidase activity, etc. Category09 is 'Compositional proteins and biogenesis', which includes proteins involved in lipopolysaccharide biosynthetic process, phage tail and capsid proteins and their biogenesis. Category10 is 'transcription and regulation of gene expression', which includes mainly transcription factors, repressors and other regulators of gene expression activity. Category11 is 'translation, ribosomal structure and biogenesis', which includes t-RNA synthetase and ligase activity. Category12 is 'Toxins and detoxification', which includes proteins involved in toxins, post-segregating killing and detoxification of mercury ion. Category13 is 'Phage function unknown'. Category14 is 'Plasmid function unknown'. Category15 is 'Unknown', which includes gene families that have no annotation. Category16 is 'Others', which includes all gene families of other low abundance gene functions in the gut samples.

# 2 Supplementary Figures and Tables

## 2.1 Supplementary Figures

**
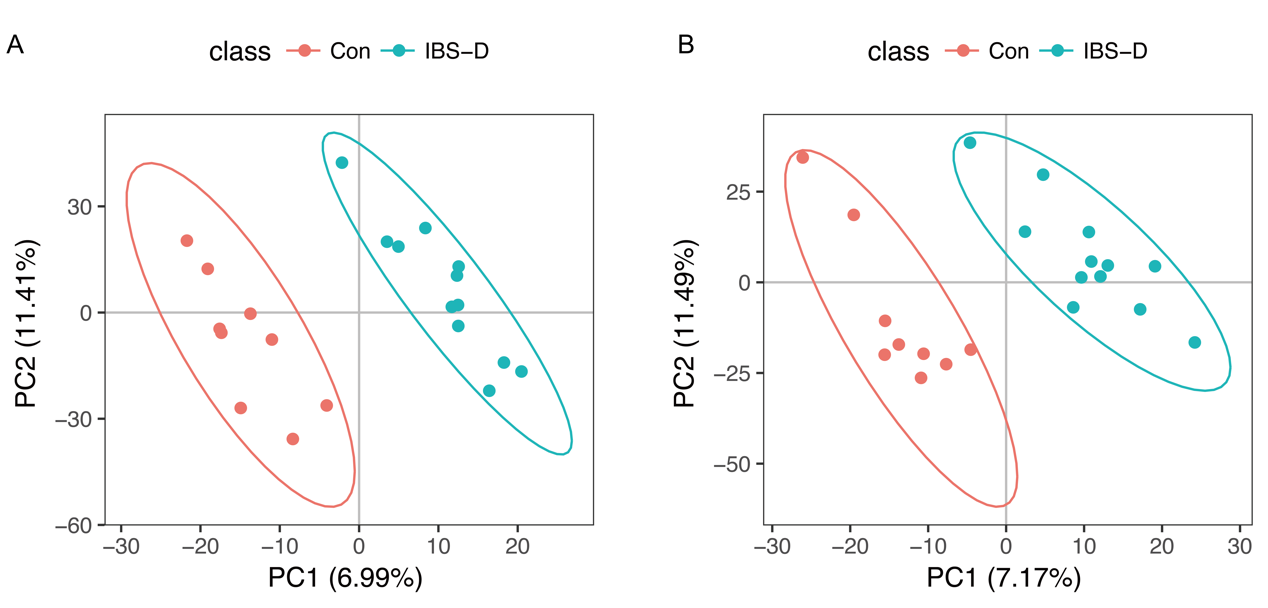
**

**Figure S1.** Partial least squares discriminant analysis plot showing discrimination of patients with irritable bowel syndrome from healthy controls using their serum (A) negative metabolomic profile and (B) positive metabolic ions.


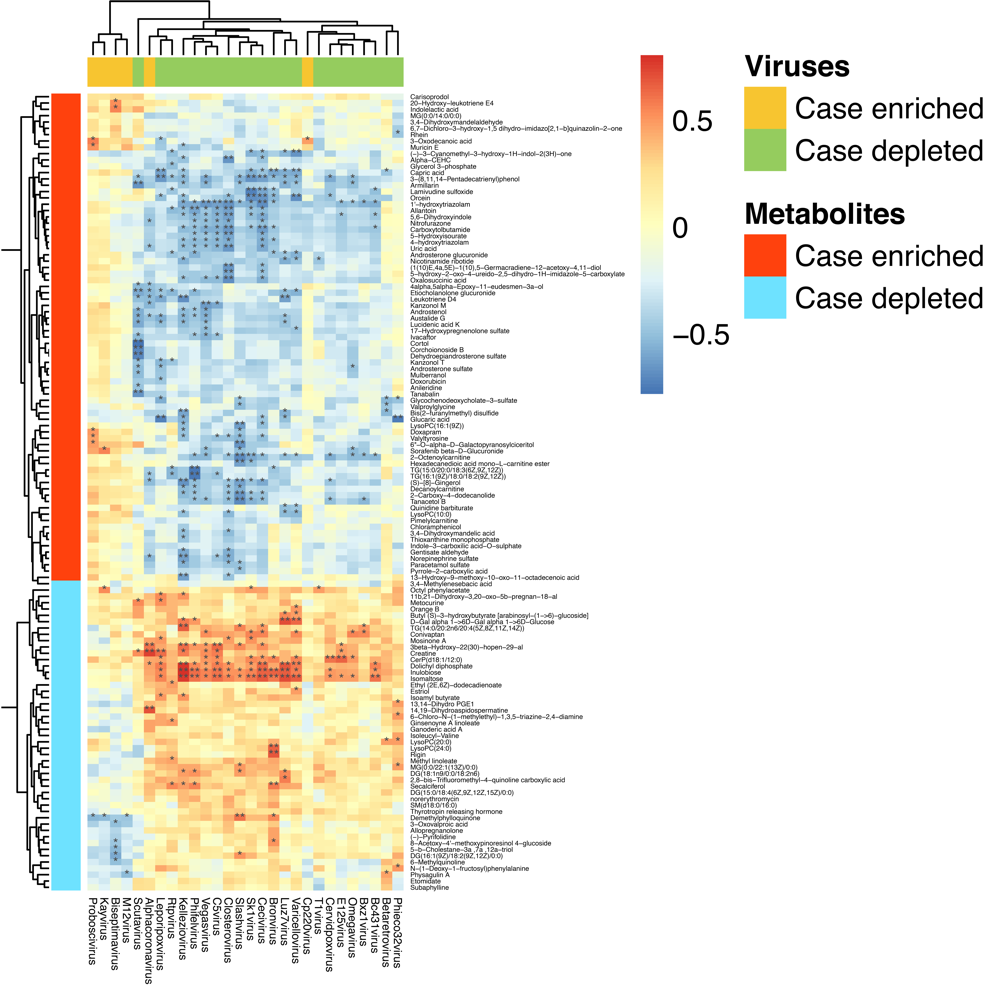


**Figure S2.** Heatmap of the Spearman correlations between significantly different virus genera (column) and significantly different metabolic ions (row). There were 51 metabolic ions depleted and 85 enriched in IBS group (*t-*test, *P* ≤ 0.05). The significances of correlations were labelled with ‘*’ (FDR *P* < 0.05) and ‘**’ (FDR *P* < 0.01).

**
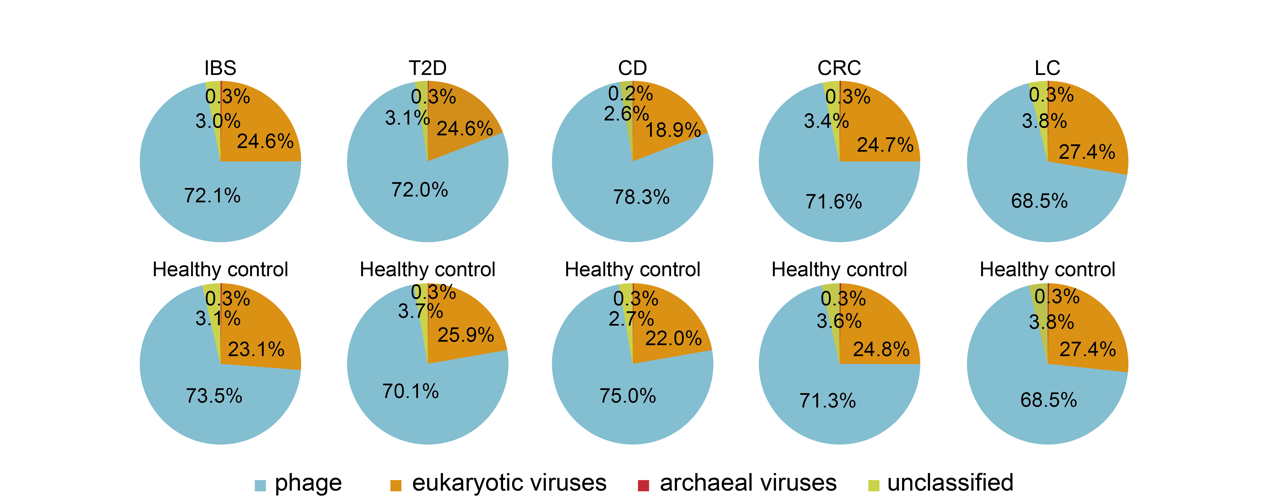
**

**Figure S3.** Compositions of gut viruses grouped by the host superkingdom.

**
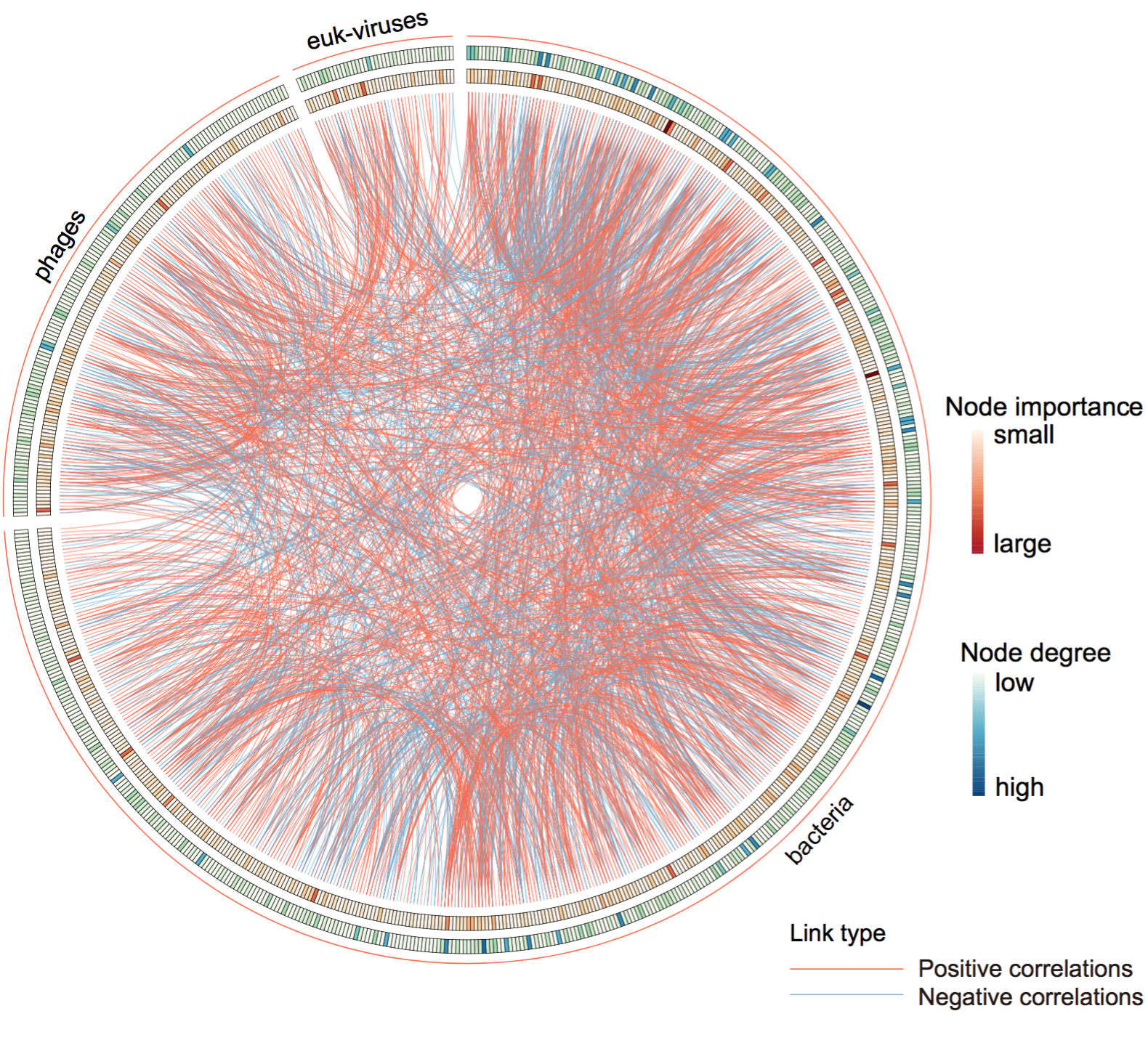
**

**Figure S4.** T2D specific co-abundance network of gut microbiome in genus level.

**
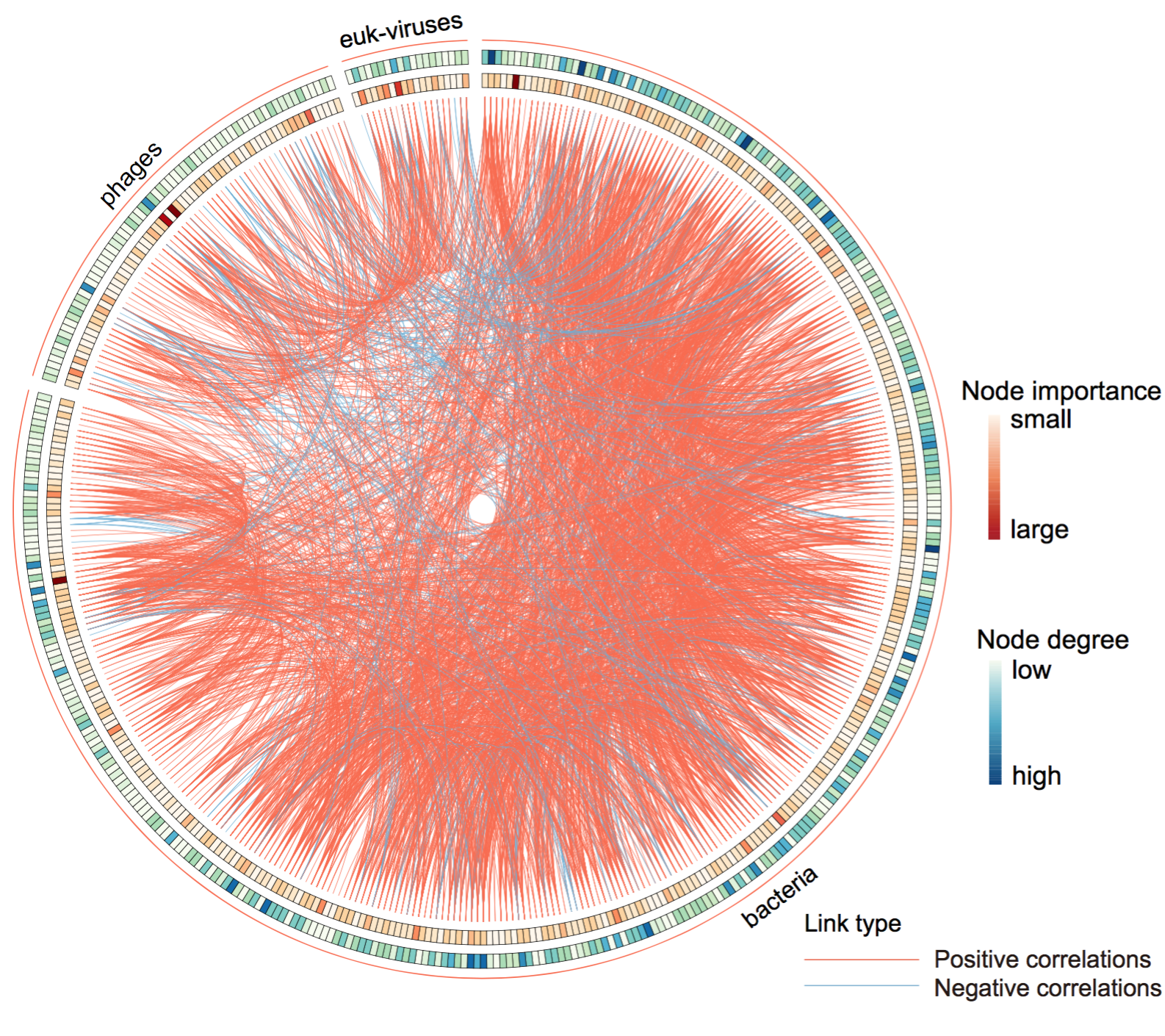
**

**Figure S5.** CD specific co-abundance network of gut microbiome in genus level.

**
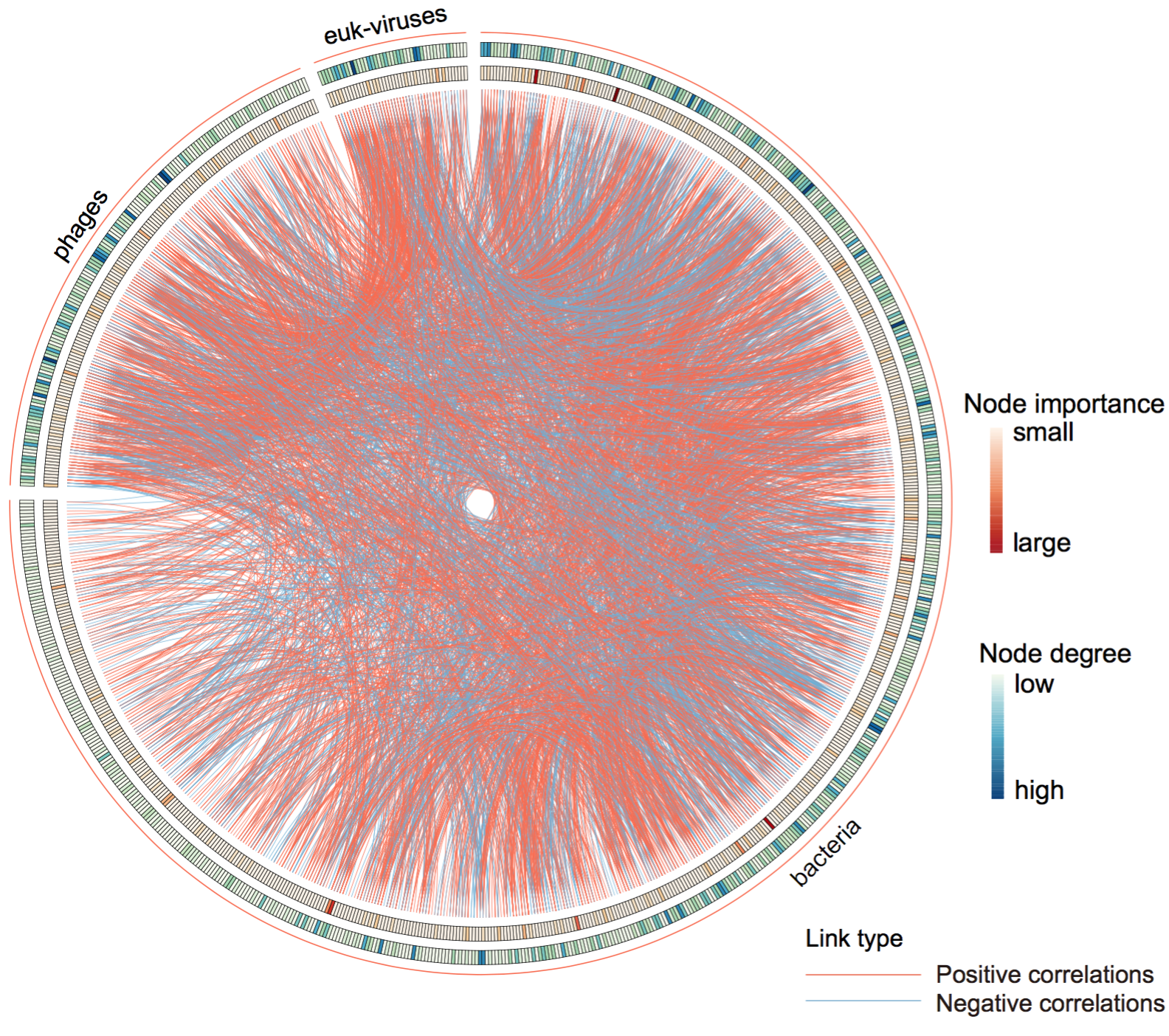
**

**Figure S6.** CRC specific co-abundance network of gut microbiome in genus level.

**
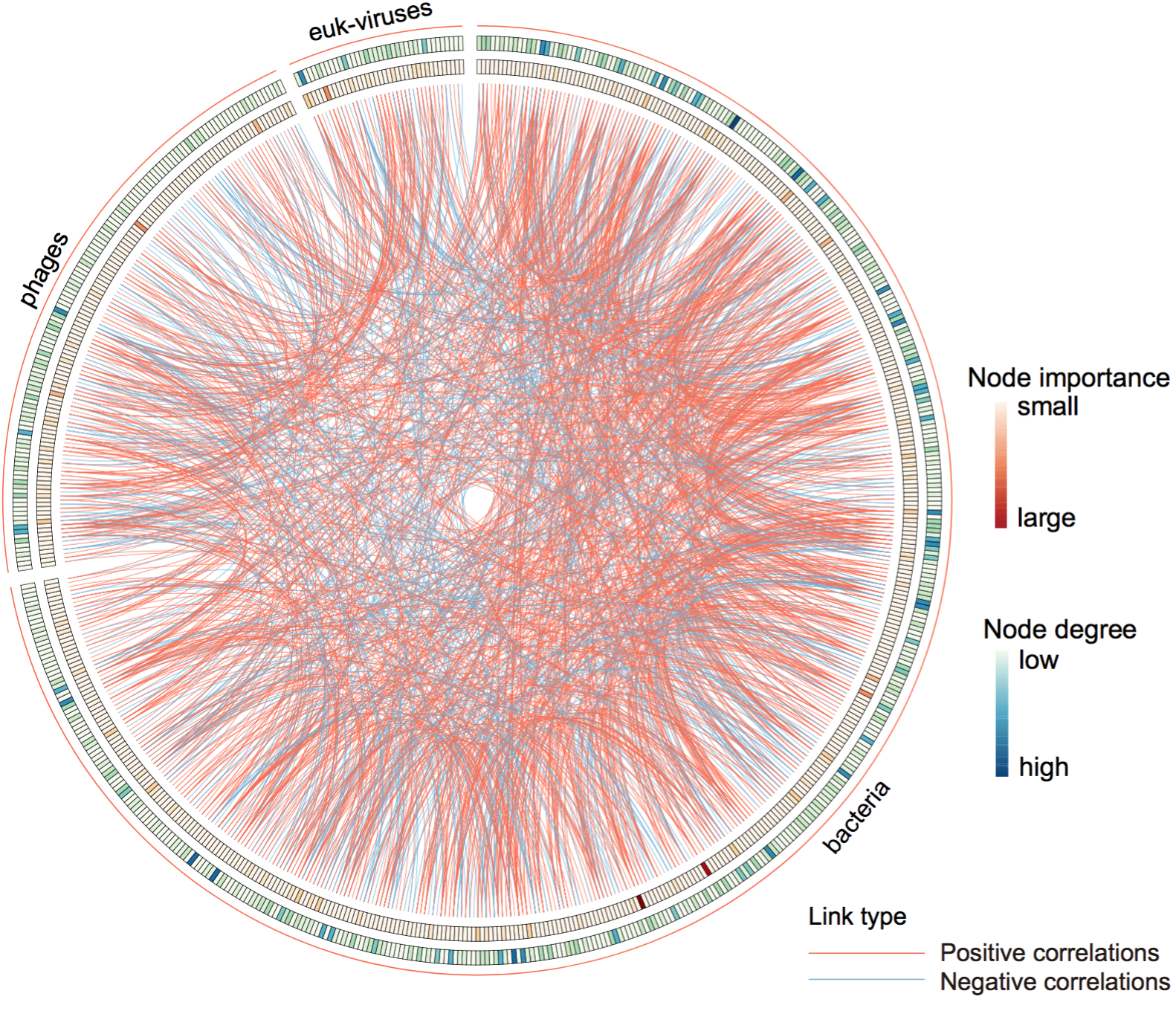
**

**Figure S7.** LC specific co-abundance network of gut microbiome in genus level.

**
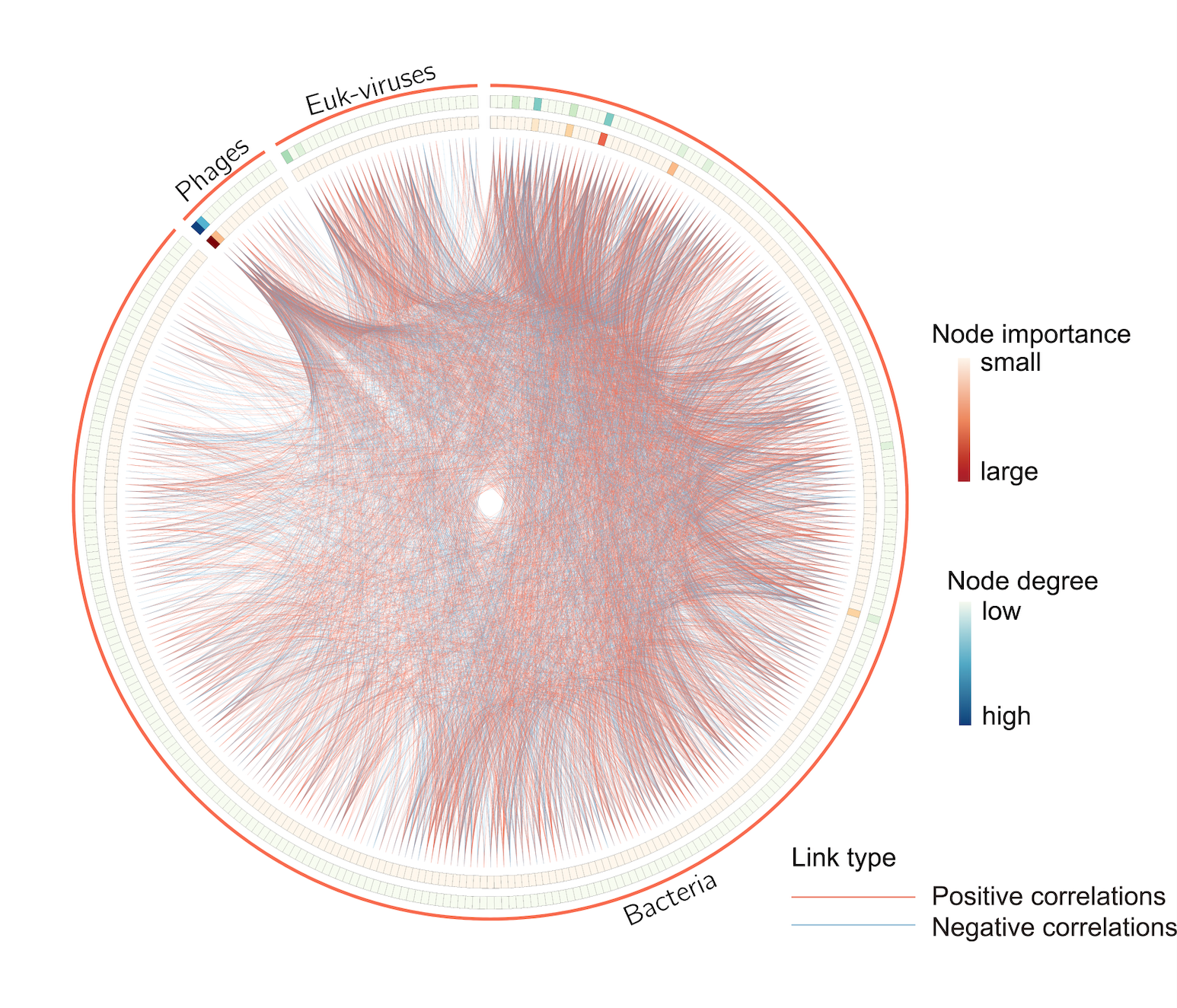
**

**Figure S8.** Pan network of five healthy-specific networks in family level.

**
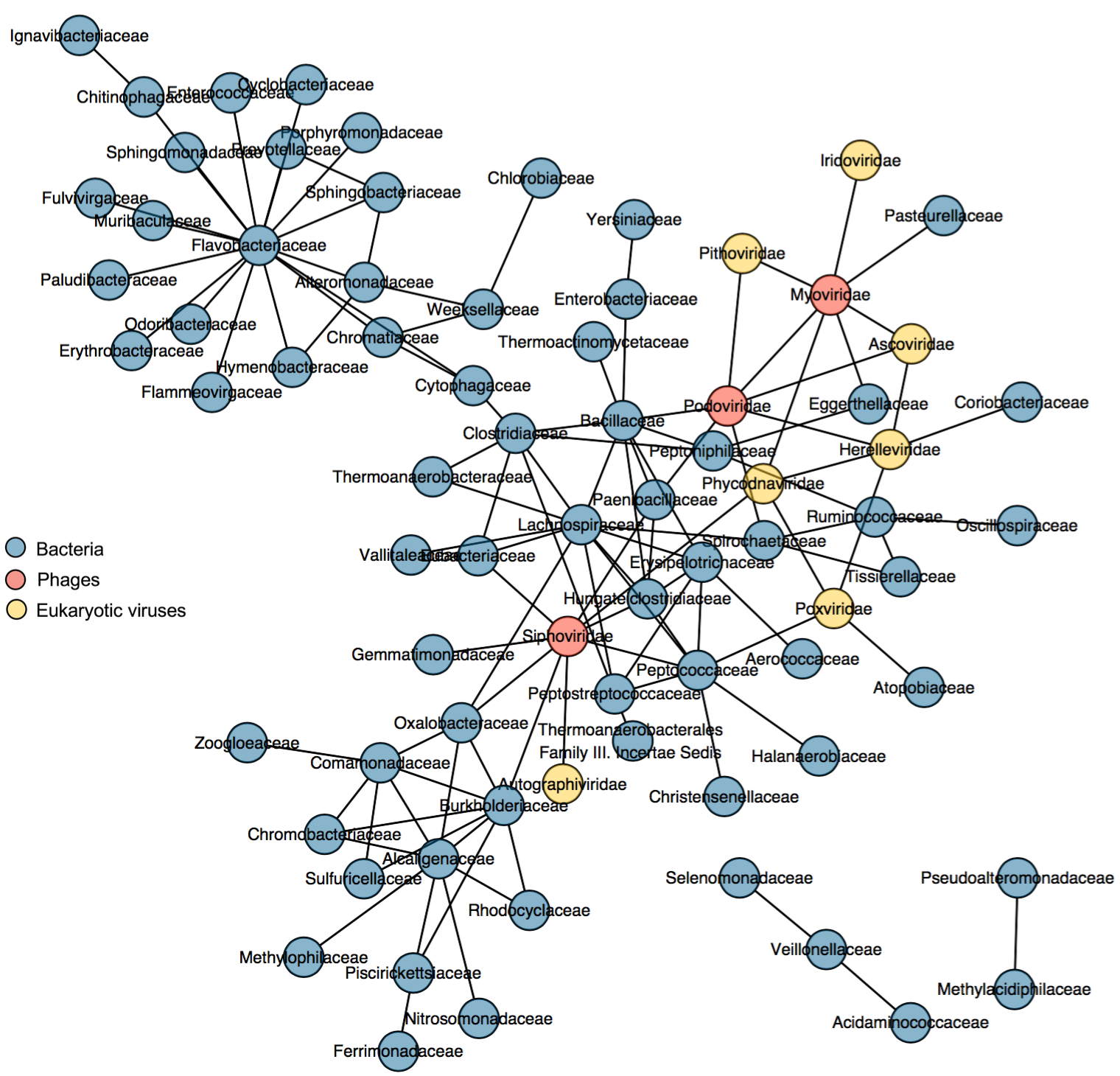
**

**Figure S9.** Core network of disease-specific networks in family level. *Siphoviridae*, *Myoviridae*, *Podoviridae* are in the hub positions.


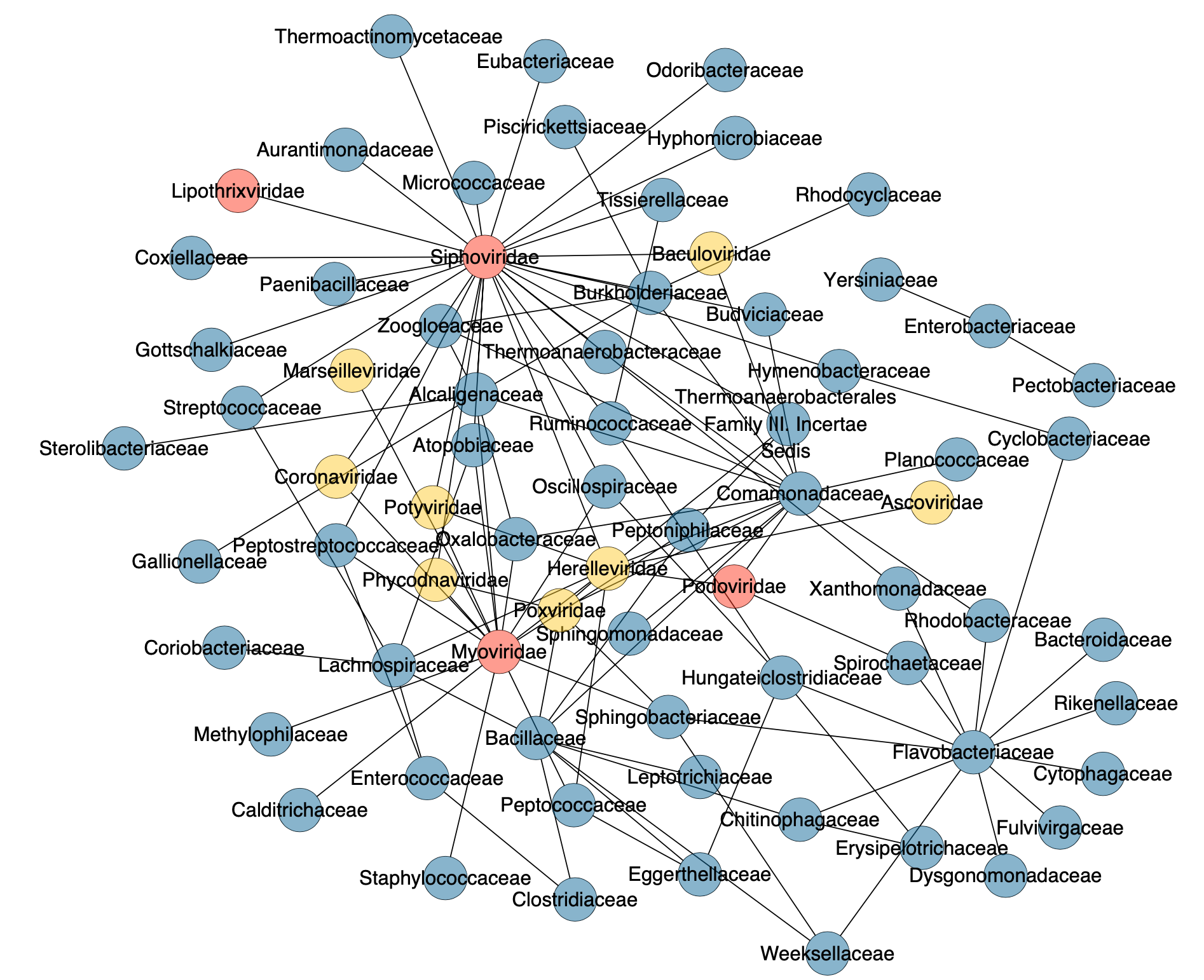


**Figure S10.** Core network of healthy-specific networks in family level. *Siphoviridae*, *Myoviridae*, *Podoviridae* are in the hub positions.


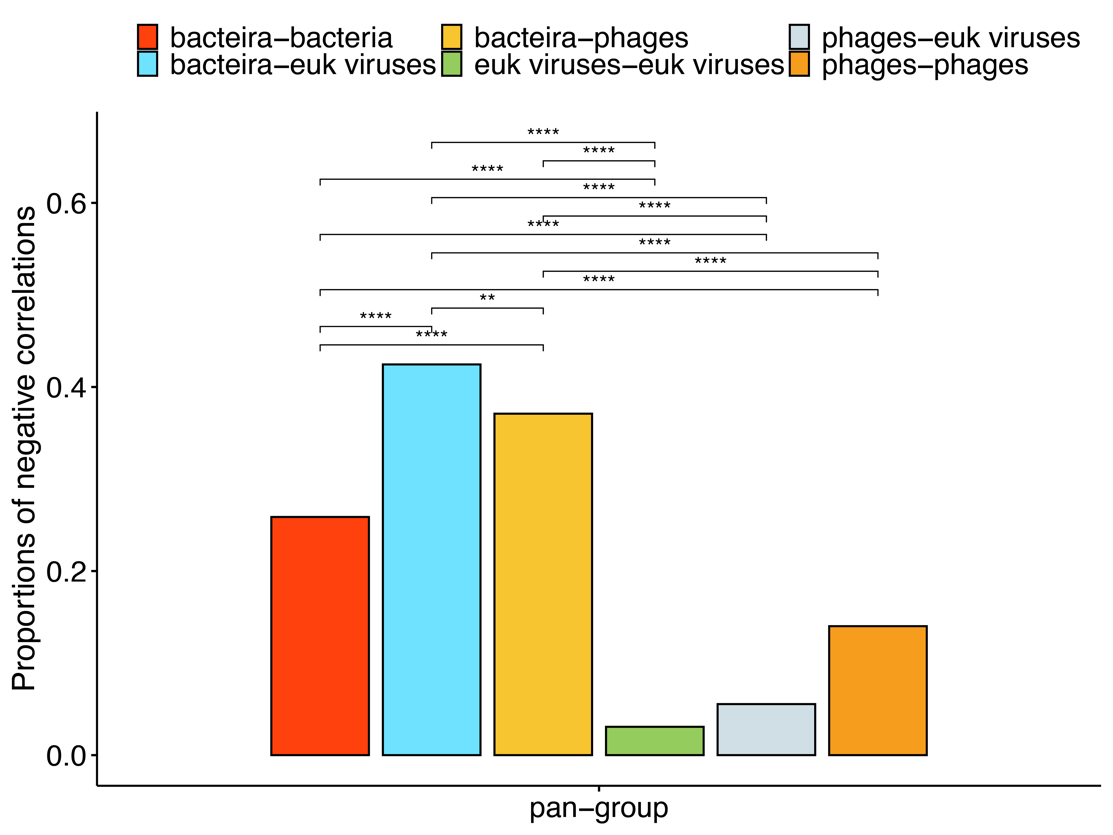


**Figure S11.** Histogram of negative correlation ratios within and between three classes of nodes: bacteria, phages, and eukaryotic viruses in the pan-disease-specific network (‘*’: FDR *P* < 0.05, ‘**’: FDR *P* < 0.01, ‘***’: FDR *P* < 0.001, ‘****’: FDR *P* < 0.0001).

**
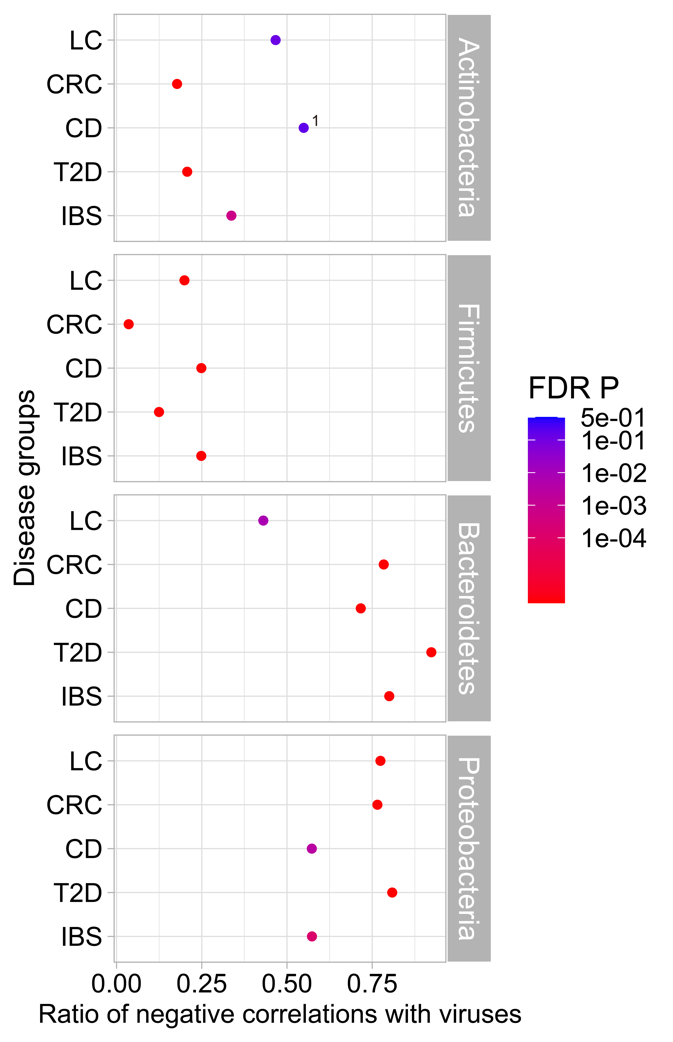
**

**Figure S12.** Ratio of negative correlations of four bacterial phyla that link to viruses in healthy-specific networks. Points with label '1' denote FDR *P* > 0.05. The *p*-values were calculated two-sided, so the negative correlation ratio close to 0 or 1 and adjusted FDR *P* < 0.05 means the positive correlation ratio or negative correlation ratio (respectively) was enriched in the relationships between that bacteria with viruses.

**
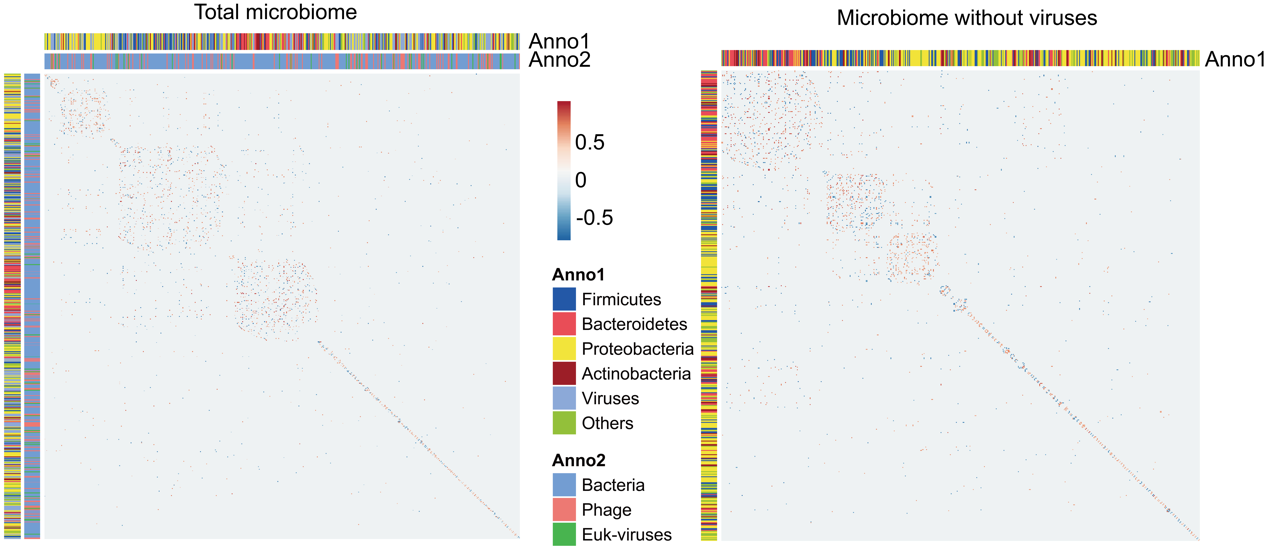
**

**Figure S13.** Heatmaps showing the modularity of the T2D-specific co-abundance network. The colors in the heatmaps represent SparCC correlations.

**
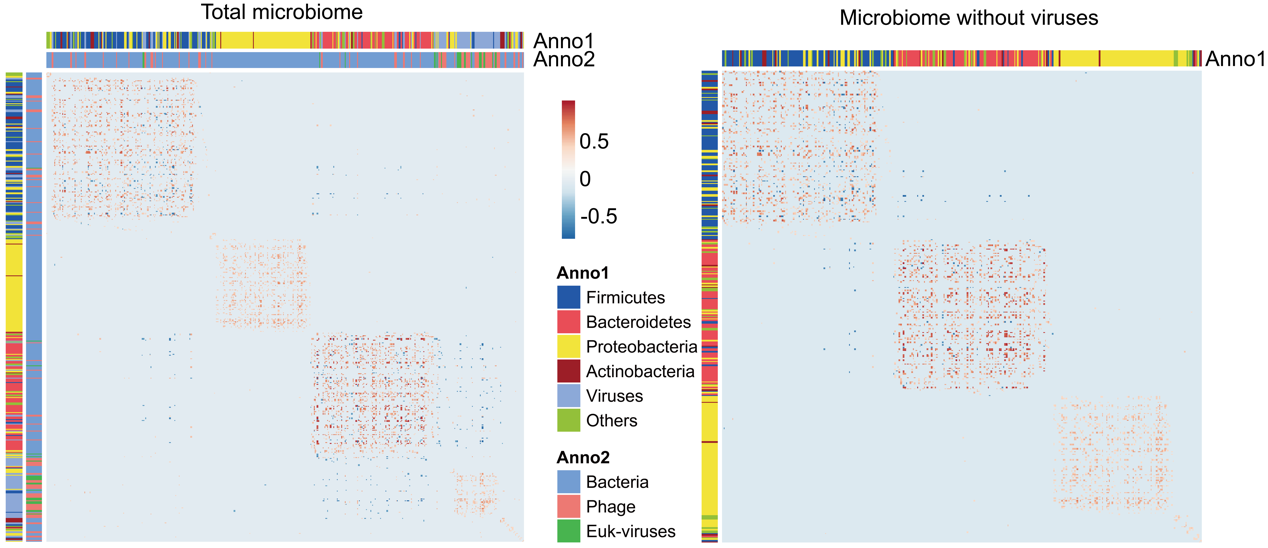
**

**Figure S14.** Heatmaps showing the modularity of the CD-specific co-abundance network. The colors in the heatmaps represent SparCC correlations.

**
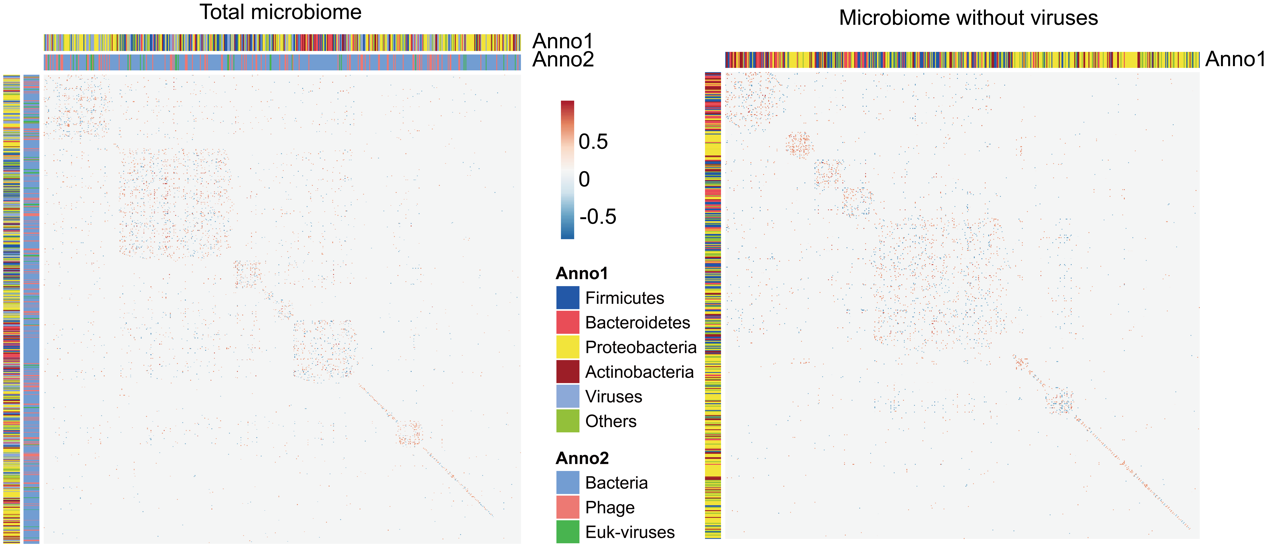
**

**Figure S15.** Heatmaps showing the modularity of the CRC-specific co-abundance network. The colors in the heatmaps represent SparCC correlations.

**
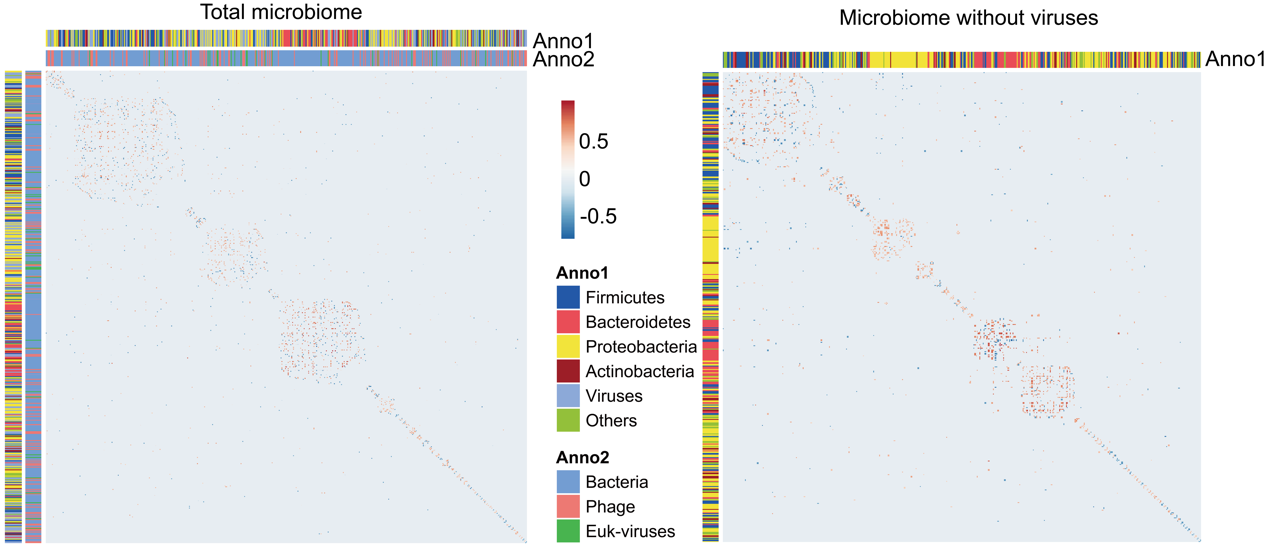
**

**Figure S16.** Heatmaps showing the modularity of the LC-specific co-abundance network. The colors in the heatmaps represent SparCC correlations.

## 2.2 Supplementary Tables

**Table S8**. Mean degrees of different nodes in each network. 'Euk-viruses' stand for eukaryotic viruses.

|  | | Dataset | Bacteria | Phages | Euk-viruses |
| --- | --- | --- | --- | --- | --- |
| Disease-specific network | IBS | | 11.4 | 11.4 | 16.7 |
|  | T2D | | 5.18 | 3.72 (W = 45485, *P* = 0.001) | 3.45 (W = 14416, *P* = 0.007) |
|  | CD | | 14.4 | 7.06 (W = 16495, *P* < 0.001) | 10.5 |
|  | CRC | | 8.10 | 8.97 | 11.4 (W = 10298, *P* = 0.001) |
|  | LC | | 4.93 | 3.40 (W = 36293, *P* < 0.001) | 4.11 |

| Healthy-specific network | IBS | 5.28 | 5.17 | 5.36 |
| --- | --- | --- | --- | --- |
|  | T2D | 8.45 | 9.37 | 12.8 (W = 8079.5, *P* = 0.01) |
|  | CD | 4.95 | 4.67 | 5.28 |
|  | CRC | 10.5 | 9.46 | 10.4 |
|  | LC | 7.80 | 7.06 | 7.72 |

Numbers in red means the value is significantly greater than the value of 'Bacteria' in the same row, while number in blue means the value is smaller than the value of 'Bacteria' in the same row. Mann-Whitney U test was used to test the significance.

**Table S11**. 16 functional categories of viral gene families.

| Category number | Description | Protein families included |
| --- | --- | --- |
| Categoty01 | Integration and recombination | This category mainly includes function of phage DNA integration, tyrosine-based recombinase activity, site-specific DNA recombination, DDE-based recombinase activity and transpositional DNA recombination. |
| Categoty02 | DNA/RNA replication and repair | This category includes proteins such as polymerase, helicase, topoisomerase, ligase activities and DNA mismatch repair proteins. |
| Categoty03 | Metabolic enzymes | This category includes generally described proteins that cannot be classified into a specific function, such as oxidoreductase activity, hydrolase activity, and modification related activity. |
| Categoty04 | Transporter activity | This category includes ATP-binding cassette (ABC) transporter, transmembrane transporter, as well as some host-related pathways such as response to drugs, detoxification to mercury ion, response to antibiotics. |
| Categoty05 | Signal transduction | This category includes two-component signal transduction system and proteins that response to stress. |
| Categoty06 | Nucleotide transport and metabolism | This category includes proteins involved in nucleotide biosynthesis and metabolism activities. |
| Categoty07 | Chaperons and secretion system | This category includes all sort of chaperons and proteins in secretion system (mainly type III and type IV secretion system). |
| Categoty08 | Phage lysis | This category includes functions related to endolysin activity, phage release by lysis activity and cell wall degrading amidase activity, *etc.* |
| Categoty09 | Compositional proteins and biogenesis | This category includes proteins involved in lipopolysaccharide biosynthetic process, phage tail and capsid proteins and their biogenesis. |
| Categoty10 | Transcription and regulation of gene expression | This category includes transcription factors, repressors and other regulators of gene expression activity. |
| Categoty11 | translation, ribosomal structure and biogenesis | This category includes t-RNA synthetase and ligase activity. |
| Categoty12 | Toxins and detoxification | This category includes proteins involved in toxins, post-segregating killing and detoxification of mercury ion. |
| Categoty13 | Phage function unknown | This category includes proteins of unknown functions of phages. |
| Categoty14 | Plasmid function unknown | This category includes proteins of unknown functions of plasmids. |
| Categoty15 | Unknown | This category includes gene families that have no GO annotation nor protein name annotation. |
| Categoty16 | Others | This category includes all gene families of other low abundance gene functions in the gut samples. |

**Table S12**. Functional enrichment of genes of key viruses in disease-specific networks based on VirGenFunD categories.

| VieGenFunD category | FDR *P* | Description | Group |
| --- | --- | --- | --- |
| category04 | 2.70 × 10^-34^ | Transporter activity | IBS |
| category05 | 3.52 × 10^-15^ | Signal transduction | IBS |
| category04 | 0 | Transporter activity | T2D |
| category07 | 7.93 × 10^-19^ | Chaperons and secretion system | T2D |
| category16 | 2.90 × 10^-11^ | Others | T2D |
| category04 | 0 | Transporter activity | CD |
| category07 | 2.02 × 10^-69^ | Chaperons and secretion system | CD |
| category03 | 0 | Metabolic enzymes | CRC |
| category16 | 0 | Others | CRC |
| category09 | 5.06 × 10^-151^ | Compositional proteins and biogenesis | CRC |
| category10 | 1.67 × 10^-103^ | Transcriptionand regulation of gene expression | CRC |
| category06 | 7.54 × 10^-86^ | Nucleotide transport and metabolism | CRC |
| category13 | 7.68 × 10^-50^ | Phage function unknown | CRC |
| category15 | 5.37 × 10^-4^ | Unknown | CRC |
| category04 | 0 | Transporter activity | LC |
| category07 | 1.11 × 10^-3^ | Chaperons and secretion system | LC |

**Table S13**. Functional enrichment of positive interacting phages with bacteria in disease-specific networks based on VirGenFunD categories.

| VieGenFunD category | FDR *P* | Description | Group |
| --- | --- | --- | --- |
| category03 | 1.88 × 10^-71^ | Metabolic enzymes | T2D |
| category09 | 3.32 × 10^-70^ | Compositional proteins and biogenesis | T2D |
| category13 | 3.99 × 10^-63^ | Phage function unknown | T2D |
| category01 | 7.24 × 10^-11^ | Integration and recombination | T2D |
| category06 | 4.52 × 10^-7^ | Nucleotide transport and metabolism | T2D |
| category10 | 0.014 | Transcription and regulation of gene expression | T2D |
| category13 | 9.14 × 10^-299^ | Phage function unknown | CD |
| category03 | 2.26 × 10^-226^ | Metabolic enzymes | CD |
| category09 | 4.32 × 10^-180^ | Compositional proteins and biogenesis | CD |
| category08 | 1.34 × 10^-128^ | Phage lysis | CD |
| category10 | 2.92 × 10^-34^ | Transcription and regulation of gene expression | CD |
| category06 | 1.61 × 10^-11^ | Nucleotide transport and metabolism | CD |
| category01 | 8.26 × 10^-6^ | Integration and recombination | CD |
| category15 | 2.65 × 10^-4^ | Unknown | CD |
| category16 | 1.27 × 10^-211^ | Others | CRC |
| category15 | 1.20 × 10^-128^ | Unknown | CRC |
| category13 | 1.16 × 10^-57^ | Phage function unknown | CRC |
| category14 | 4.68 × 10^-26^ | Plasmid function unknown | CRC |
| category08 | 7.48 × 10^-7^ | Phage lysis | CRC |
| category02 | 9.91 × 10^-6^ | DNA/RNA replication and repair | CRC |
| category10 | 1.11 × 10^-100^ | Transcription and regulation of gene expression | LC |
| category02 | 8.75 × 10^-66^ | DNA/RNA replication and repair | LC |
| category01 | 8.19 × 10^-52^ | Integration and recombination | LC |
| category08 | 1.83 × 10^-10^ | Phage lysis | LC |
| category13 | 5.75 × 10^-189^ | Phage function unknown | IBS |
| category08 | 1.33 × 10^-48^ | Phage lysis | IBS |
| category06 | 9.24 × 10^-26^ | Nucleotide transport and metabolism | IBS |
| category16 | 8.38 × 10^-18^ | Others | IBS |
| category10 | 1.00 × 10^-12^ | Transcription regulation of gene expression | IBS |
| category01 | 5.74 × 10^-8^ | Integration and recombination | IBS |

**Table S14**. Functional enrichment of negative interacting phages with bacteria in disease-specific networks based on VirGenFunD categories.

| VirGenFunD category | FDR *P* | Description | Group |
| --- | --- | --- | --- |
| category02 | 2.24 × 10^-47^ | DNA/RNA replication and repair | T2D |
| category16 | 3.53 × 10^-33^ | Others | T2D |
| category03 | 1.70 × 10^-11^ | Metabolic enzymes | T2D |
| category08 | 6.00 × 10^-4^ | Phage lysis | T2D |
| category08 | 4.62 × 10^-209^ | Phage lysis | CD |
| category16 | 8.36 × 10^-112^ | Others | CD |
| category02 | 1.23 × 10^-30^ | DNA/RNA replication and repair | CD |
| category09 | 2.98 × 10^-12^ | Compositional proteins and biogenesis | CD |
| category01 | 0 | Integration and recombination | CRC |
| category08 | 1.79 × 10^-29^ | Phage lysis | CRC |
| category13 | 4.13 × 10^-28^ | Phage function unknown | CRC |
| category16 | 3.99 × 10^-50^ | Others | LC |
| category03 | 5.99 × 10^-20^ | Metabolic enzymes | LC |
| category13 | 5.76 × 10^-12^ | Phage function unknown | LC |
| category12 | 1.23 × 10^-6^ | Toxins and detoxification | LC |
| category08 | 6.87 × 10^-3^ | Phage lysis | LC |
| category15 | 2.78 × 10^-76^ | Unknown | IBS |
| category16 | 4.37 × 10^-29^ | Others | IBS |
| category01 | 0.028 | Integration and recombination | IBS |

**Table S15**. Functional enrichment of positive interacting phages with bacteria in healthy-specific networks based on VirGenFunD categories.

| VirGenFunD category | FDR *P* | Description | Group |
| --- | --- | --- | --- |
| category10 | 5.99 × 10^-292^ | transcription and regulation of gene expression | T2D |
| category01 | 2.60 × 10^-68^ | Integration and recombination | CD |
| category06 | 7.76 × 10^-6^ | Nucleotide transport and metabolism | CD |
| category02 | 1.38 × 10^-4^ | DNA/RNA replication and repair | CD |
| category09 | 0.030 | Compositional proteins and biogenesis | CD |
| category15 | 1.65 × 10^-121^ | Unknown | CRC |
| category16 | 2.66 × 10^-75^ | Others | CRC |
| category14 | 1.77 × 10^-67^ | Plasmid function unknown | CRC |
| category01 | 4.29 × 10^-16^ | Integration and recombination | CRC |
| category13 | 4.09 × 10^-7^ | Phage function unknown | CRC |
| category04 | 0 | Transporter activity | LC |
| category05 | 3.69 × 10^-37^ | Signal transduction | LC |
| category07 | 1.17 × 10^-8^ | Chaperons and secretion system | LC |
| category10 | 7.49 × 10^-196^ | transcription and regulation of gene expression | IBS |
| category03 | 1.35 × 10^-172^ | Metabolic enzymes | IBS |
| category01 | 5.17 × 10^-93^ | Integration and recombination | IBS |
| category13 | 2.14 × 10^-20^ | Phage function unknown | IBS |
| category12 | 7.20 × 10^-3^ | Toxins and detoxification | IBS |
| category09 | 0.0367 | Compositional proteins and biogenesis | IBS |

**Table S16**. Functional enrichment of negative interacting phages with bacteria in healthy-specific networks based on VirGenFunD categories.

| VirGenFunD category | FDR *P* | Description | Group |
| --- | --- | --- | --- |
| category02 | 2.42 × 10^-54^ | DNA/RNA replication and repair | T2D |
| category13 | 1.46 × 10^-9^ | Phage function unknown | T2D |
| category08 | 1.57 × 10^-6^ | Phage lysis | T2D |
| category06 | 8.32 × 10^-5^ | Nucleotide transport and metabolism | T2D |
| category15 | 0.027 | Unknown | T2D |
| category10 | 4.92 × 10^-60^ | transcription and regulation of gene expression | CD |
| category01 | 2.04 × 10^-4^ | Integration and recombination | CD |
| category14 | 0.018 | Plasmid function unknown | CD |
| category12 | 0.045 | Toxins and detoxification | CD |
| category06 | 4.79 × 10^-120^ | Nucleotide transport and metabolism | CRC |
| category01 | 1.99 × 10^-62^ | Integration and recombination | CRC |
| category10 | 3.40 × 10^-40^ | transcription and regulation of gene expression | CRC |
| category03 | 4.61 × 10^-19^ | Metabolic enzymes | CRC |
| category15 | 4.22 × 10^-9^ | Unknown | CRC |
| category13 | 3.00 × 10^-4^ | Phage function unknown | CRC |
| category14 | 1.28 × 10^-3^ | Plasmid function unknown | CRC |
| category09 | 3.04 × 10^-4^ | Compositional proteins and biogenesis | CRC |
| category01 | 7.46 × 10^-43^ | Integration and recombination | LC |
| category03 | 2.08 × 10^-41^ | Metabolic enzymes | LC |
| category10 | 4.42 × 10^-5^ | transcription and regulation of gene expression | LC |
| category02 | 6.67 × 10^-5^ | DNA/RNA replication and repair | LC |
| category12 | 7.72 × 10^-3^ | Toxins and detoxification | LC |
| category04 | 0 | Transporter activity | IBS |
| category05 | 4.11 × 10^-56^ | Signal transduction | IBS |
| category07 | 1.11 × 10^-38^ | Chaperons and secretion system | IBS |

**Table S17**. Functional enrichment of interacting eukaryotic viruses with bacteria in disease-specific networks based on VirGenFunD categories.

| VirGenFunD category | FDR *P* | Description | Group |
| --- | --- | --- | --- |
| Category05 | 0 | Signal transduction | T2D |
| Category03 | 2.20 × 10^-170^ | Metabolic enzymes | T2D |
| Category03 | 0 | Metabolic enzymes | CD |
| Category04 | 0 | Transporter activity | CD |
| Category16 | 3.18 × 10^-223^ | Others | CD |
| Category06 | 1.13 × 10^-10^ | Nucleotide transport and metabolism | CD |
| Category03 | 0 | Metabolic enzymes | CRC |
| Category05 | 0 | Signal transduction | CRC |
| Category04 | 5.55 × 10^-15^ | Transporter activity | CRC |
| Category05 | 0 | Signal transduction | LC |
| Category03 | 1.06 × 10^-27^ | Metabolic enzymes | LC |
| Category04 | 3.41 × 10^-17^ | Transporter activity | LC |
| Category05 | 0 | Signal transduction | IBS |
| Category03 | 1.42 × 10^-215^ | Metabolic enzymes | IBS |
| Category04 | 1.84 × 10^-8^ | Transporter activity | IBS |

**Table S18**. Functional enrichment of interacting eukaryotic viruses with bacteria in healthy-specific networks based on VirGenFunD categories.

| VirGenFunD category | FDR *P* | Description | Group |
| --- | --- | --- | --- |
| Category05 | 0 | Signal transduction | IBS |
| Category16 | 2.74 × 10^-30^ | Others | IBS |
| Category02 | 2.43 × 10^-14^ | DNA/RNA replication and repair | IBS |
| Category07 | 4.09 × 10^-15^ | Chaperons and secretion system | IBS |
| Category10 | 6.34 × 10^-4^ | Transcription and regulation of gene expression | IBS |
